# Characterisation of ovine bone marrow-derived stromal cells (oBMSC) and evaluation of chondrogenically induced micro-pellets for cartilage tissue repair in vivo

**DOI:** 10.1186/s13287-020-02045-3

**Published:** 2021-01-07

**Authors:** K. Futrega, E. Music, P. G. Robey, S. Gronthos, R. Crawford, S. Saifzadeh, T. J. Klein, M. R. Doran

**Affiliations:** 1 Centre for Biomedical Technologies (CBT), Queensland University of Technology (QUT), Brisbane, Queensland Australia; 2 National Institute of Dental and Craniofacial Research (NIDCR), National Institutes of Health (NIH), Bethesda, Maryland USA; 3 Translational Research Institute (TRI), Brisbane, Queensland Australia; 4 School of Biomedical Sciences, Queensland University of Technology (QUT), Brisbane, Queensland Australia; 5 Adelaide Medical School, Faculty of Health and Medical Sciences, University of Adelaide, Adelaide, South Australia Australia; 6 Mater Research Institute – University of Queensland (UQ), Translational Research Institute (TRI), Brisbane, Queensland Australia

**Keywords:** Bone marrow stromal cells, Mesenchymal stem cells, Cartilage, Osteoarthritis, Chondrogenesis, Differentiation, Hypertrophy, Oxygen, Micro-pellet

## Abstract

**Abstract:**

Bone marrow stromal cells (BMSC) show promise in cartilage repair, and sheep are the most common large animal pre-clinical model.

**Objective:**

The objective of this study was to characterise ovine BMSC (oBMSC) in vitro, and to evaluate the capacity of chondrogenic micro-pellets manufactured from oBMSC or ovine articular chondrocytes (oACh) to repair osteochondral defects in sheep.

**Design:**

oBMSC were characterised for surface marker expression using flow cytometry and evaluated for tri-lineage differentiation capacity. oBMSC micro-pellets were manufactured in a microwell platform, and chondrogenesis was compared at 2%, 5%, and 20% O_2_. The capacity of cartilage micro-pellets manufactured from oBMSC or oACh to repair osteochondral defects in adult sheep was evaluated in an 8-week pilot study.

**Results:**

Expanded oBMSC were positive for CD44 and CD146 and negative for CD45. The common adipogenic induction ingredient, 3-Isobutyl-1-methylxanthine (IBMX), was toxic to oBMSC, but adipogenesis could be restored by excluding IBMX from the medium. BMSC chondrogenesis was optimal in a 2% O_2_ atmosphere. Micro-pellets formed from oBMSC or oACh appeared morphologically similar, but hypertrophic genes were elevated in oBMSC micro-pellets. While oACh micro-pellets formed cartilage-like repair tissue in sheep, oBMSC micro-pellets did not.

**Conclusion:**

The sensitivity of oBMSC, compared to human BMSC, to IBMX in standard adipogenic assays highlights species-associated differences. Micro-pellets manufactured from oACh were more effective than micro-pellets manufactured from oBMSC in the repair of osteochondral defects in sheep. While oBMSC can be driven to form cartilage-like tissue in vitro, the effective use of these cells in cartilage repair will depend on the successful mitigation of hypertrophy and tissue integration.

**Supplementary information:**

The online version contains supplementary material available at 10.1186/s13287-020-02045-3.

## Introduction

Despite considerable investment into bone marrow-derived stromal cells (BMSC, sometimes referred to as “mesenchymal stem cells”) as a cell source for cartilage defect repair, thus far, no BMSC-based therapies have successfully passed the regulatory and efficacy hurdles required for clinical approval [1]. A major efficacy limitation for BMSC-derived engineered cartilage is the observed hypertrophic conversion of these cells when transplanted in vivo in mice, resulting in remodelled, mineralised bone-like tissue [2, 3].

The majority of pre-clinical experimentation is performed in small animal models such as mice, rats, or rabbits [4, 5]. While some orthopaedic studies are performed using the joints of these small animals as models, these joints are anatomically different and experience reduced mechanical forces relative to human joints. Additionally, the thin cartilage in small animal models is capable of regeneration, which differs from cartilage in human joints [4]. In some studies that use immunocompromised small animals, human tissue is implanted at ectopic sites, typically in subcutaneous pouches on the backs of mice [2, 3]. While this allows for the transplantation of human cells, the implant sites are dissimilar to human joints, as they do not provide mechanical load, and this site is more vascular than joint capsules. Large animal models such as sheep, pigs, goats, or horses are more appropriate for orthopaedic studies due to their joint anatomy, and healing properties being more similar to that of humans [6]. However, unlike immunocompromised small animal models, these immune competent large animal models require that species-matched cells be used. Therefore, large animal models need to be characterised independently to determine their suitability as models for cellular therapies. While sheep are the most commonly used large animal for the study of cartilage repair [6], the biology of sheep or ovine BMSC (oBMSC) remains poorly characterised relative to human BMSC (hBMSC).

Our team has developed micro-pellet models to characterise and optimise in vitro hBMSC chondrogenesis [3, 7, 8]. Pellet models for studying BMSC chondrogenesis were first described by Johnstone et al. in 1998 [9]. While the traditional pellet model mimics aspects of developmental mesenchymal condensation, these pellets, formed from ~ 2 × 10^5^ cells each, have a large diameter and suffer steep diffusion gradients and radial tissue heterogeneity [8]. By contrast, micro-pellets can be formed from fewer cells (5 × 10^3^ each), resulting in reduced diffusion gradients and more homogeneous tissue. Hereafter, traditional pellet cultures assembled from several hundred thousand cells will be referred to as macro-pellets, while smaller pellets will be referred to as micro-pellets. Previous data demonstrate that micro-pellets enable characterisation and optimisation of cell culture variables, such as optimal oxygen concentration [3] or growth factor exposure time [7], that are obfuscated by the radial heterogeneity suffered by traditional macro-pellet models. In addition to being an excellent tissue culture model, micro-pellets could theoretically be used as building blocks to fill cartilage defects and facilitate repair [3, 10]. To expedite high-throughput micro-pellet manufacture, we developed a microwell culture platform called the Microwell-mesh [3]. The Microwell-mesh has a nylon mesh bonded over an array of microwells. The mesh openings are large enough to allow a single cell suspension to be centrifuged through the mesh, and into the microwells. Within the first 3 h of culture, cells self-assemble into micro-pellets, becoming too large to escape back through the mesh; thus, they are retained in discrete microwells during weeks of culture manipulation. This feature of the Microwell-mesh allows hundreds to thousands of micro-pellets to be efficiently cultured simultaneously, facilitating large-scale experimentation with micro-pellets that would otherwise be tedious or costly.

In this study, we aimed to optimise the chondrogenic culture conditions for oBMSC using the micro-pellet model and to evaluate the use of micro-pellets as building blocks to repair osteochondral defects in sheep. We used the Microwell-mesh platform to manufacture micro-pellets formed from 5000 oBMSC each and compared them to traditional macro-pellets manufactured from 200,000 oBMSC each. Chondrogenic induction was performed in atmospheres containing 2%, 5%, or 20% O_2_, to better characterise this important chondrogenic factor [8, 11–13]. The resulting cartilage-like tissue was evaluated based on glycosaminoglycan (GAG) accumulation, histological analysis, and gene expression. A pilot study was then performed to evaluate the capacity of autologous micro-pellets, manufactured from either oBMSC or expanded ovine articular chondrocytes (oACh), to repair osteochondral defects formed in the stifle joint of adult Merino sheep. Two sheep received oBMSC micro-pellets, while another two sheep received oACh micro-pellets. Each sheep had three replicate defects, 6 mm in diameter and 1.5 mm deep. Engineered constructs were assembled during the surgery using a mould to cast a 6-mm diameter by 1.5 mm deep layer of micro-pellets, set with Baxter Tisseel fibrin glue, on a CelGro™ collagen scaffold, and fixed in place with sutures. The pilot study was terminated 8 weeks later, and defect infill was characterised via histology.

## Materials and methods

### Live animal anaesthesia and non-surgical procedures

All procedures were approved by the Queensland University of Technology (QUT) Animal Ethics Committee (approval number 1500001091). For this study, a total of seven skeletally mature sheep were used. The first three sheep (one accessed through tissue sharing, and two associated with this ethics approval) were used to collect bone marrow aspirates for in vitro studies. For the cartilage repair studies using autologous cells, four male sheep were used, two were assigned to the oBMSC micro-pellet group and two were assigned to the oACh micro-pellet group. Surgical procedures were performed under general anaesthesia and aseptic conditions and using a bimodal analgesics regime. For surgical knee procedures, analgesia was provided just prior to surgery with slow release transdermal fentanyl patches (2–3 mg/kg/h). A first patch was applied just prior to surgery and two more patches were applied in 3-day intervals post-surgery. Trisoprim (1 mL/30 kg/day) was administered postoperatively for 2 days to prevent bacterial infection. For euthanasia, sheep were injected in the external jugular with 100 mg/kg Pentobarbital sodium. These procedures were performed by a veterinary surgeon and team at the QUT Medical Engineering Research Facility in Brisbane, Australia. Detailed methods are available upon request from the senior corresponding author.

### Bone marrow aspiration and BMSC culture

Five different sheep were used for oBMSC in this study. In preliminary optimisation, donors oBMSC 1, oBMSC 2 and oBMSC 3 were used. In the cartilage defect repair studies, oBMSC 4 and oBMSC 5 were used. Bone marrow aspiration from the iliac crest of sheep was performed according to previously described and well-established methods [14]. Briefly, an 11-guage Jamshidi needle was used to aspirate ~ 30 mL of bone marrow from the iliac crest into a 35-mL syringe containing 5 mL of heparin (1000 IU/mL). Heparinised bone marrow aspirates were diluted in cell culture medium at a ratio of 5 mL aspirate to 25 mL medium. Growth medium consisted of low glucose Dulbecco’s modified Eagle’s medium (LG-DMEM; Thermo Fisher Scientific) supplemented with 10% fetal bovine serum (FBS; Thermo Fisher Scientific), 10 ng/mL fibroblast growth factor-1 (FGF-1; Peprotech), 5 μg/mL porcine heparin sodium salt (Sigma-Aldrich), and 100 U/mL penicillin/streptomycin (PenStrep; Thermo Fisher Scientific). Cells were seeded into Nunc™ T175 cm^2^ tissue culture flasks (Thermo Fisher Scientific) and incubated overnight in a normoxic incubator (20% O_2_) with 5% CO_2_ at 37 **°**C. Following overnight incubation, media was exchanged to remove non-adherent cells. Cultures were moved to a hypoxic incubator (2% O_2_, 5% CO_2_) following the 24-h attachment period. Adherent cells were cultured until 80% confluency, enzymatically harvested (0.25% Trypsin/EDTA, Thermo Fisher Scientific), and reseeded at 1500 cells/cm^2^ in new T175 flasks. Culture medium was exchanged twice weekly. Cells were cryopreserved at low passage (P0-P2) in 90% FBS and 10% DMSO (Sigma-Aldrich).

hBMSC isolation and expansion was performed as previously described [5]. Bone marrow aspirates were collected from the iliac crest of fully informed and consenting healthy human volunteer donors. The Mater Health Services Human Research Ethics Committee and the Queensland University of Technology Human Ethics Committee approved aspirate collection (Ethics number: 1541A). hBMSC were cultured in the same way as oBMSC.

### Flow cytometry characterisation

For flow cytometry analysis, oBMSC were stained with the following anti-human antibodies: CD34-APC, CD90-FITC, CD73-APC, CD105-PE, CD146-APC, CD271-PE, HLA-DR-PE, and isotype controls (Miltenyi Biotec). Additionally, the following anti-sheep antibodies were used: CD45-FITC and CD44-FITC (Bio-Rad). Cells were stained as per the manufacturers’ instructions, and analysis was completed using an LSR II flow cytometer (BD Biosciences). In this analysis, we also included sheep bone marrow mononuclear cells (MNC) and hBMSC. These cell populations served as controls for antibody performance against a common hBMSC population, and for sheep MNC, where haematopoietic cells (CD45^+^) would be expected. Data were analysed using FlowJo software, version 10 (BD Biosciences).

### Articular cartilage biopsy and oACh culture

Two oACh donors (oACh 1 and oACh 2) were used in the cartilage defect repair component of this study. Articular cartilage biopsies were harvested from the femoral trochlear grooves. The knee joint capsule was accessed via a medial parapatellar incision (~ 5 cm). The patella was displaced laterally and retained in a proximal position with small wound retractors. The joint capsule was opened to expose the femoral trochlear groove. Five thin (~ 1 mm thick and 4–6 mm wide) shavings of articular cartilage were aseptically removed with a scalpel and transferred to a sterile specimen jar containing LG-DMEM with PenStrep and 1X Antibiotic-Antimycotic (Anti-Anti; Thermo Fisher Scientific). The biopsied tissues were placed in a Petri dish and chopped up coarsely. The tissue was resuspended in 10 mL of digestion solution containing type II clostridial collagenase (~ 430 U/mL; Sigma-Aldrich), PenStrep, and Antibiotic-Antimicotic (Gibco) and incubated overnight in a normoxic incubator (20% O_2_) with 5% CO_2_ at 37 **°**C. Following digestion, the cells were washed twice by centrifugation (500×*g*) with LG-DMEM containing 10% FBS. oACh were grown in a hypoxic (2% O_2_) incubator in growth medium as described above for oBMSC.

### Fabrication of the Microwell-mesh culture device

The Microwell-mesh was prepared as detailed in a previous paper published by our laboratory [3]. Briefly, a ~ 4-mm layer of polydimethylsiloxane (PDMS; Dow Corning) was poured into a polystyrene negative template that had an inverted microwell pattern. PDMS was cured at 80 °C for 60 min. A wad punch was used to create discs that fit snugly into Nunc™ 6-well plates (Thermo Fisher Scientific). Individual microwells measured 2 mm × 2 mm with a depth of 0.8 mm [3]. A nylon mesh (36-μm square pore openings, part number CMN-0035, Amazon.com) was bonded to the open face of the PDMS discs with silicone glue (Selleys, Aquarium Safe). Plates were sterilised by submerging in a 70% ethanol solution for a minimum of 30 min and rinsed 3 times with phosphate-buffered saline (PBS; Thermo Fisher Scientific). Prior to cell seeding, microwells were rinsed with a sterile 5% Pluronic F-127 solution (Sigma-Aldrich) in PBS to render the PDMS surface non-adhesive and to promote cell aggregation [15, 16]. Wells were rinsed 3 times with PBS to remove excess Pluronic, and then, cells were seeded as described below.

### Osteogenic and adipogenic induction

To induce osteogenesis and adipogenesis, oBMSC were seeded at 30,000 cells/cm^2^ in standard tissue culture well plates and cultured for 21 days in the respective induction media. Osteogenic induction medium was composed of 10 mM β-glycerol phosphate, 100 nM dexamethasone (Dex), 50 μM l-ascorbic acid 2-phosphate (all from Sigma-Aldrich), 10% FBS, and PenStrep in high glucose (HG)-DMEM (Thermo Fisher Scientific). Conventional adipogenic induction medium was composed of 10 μg/mL insulin, 100 nM Dex, 25 mM indomethacin, and 3-Isobutyl-1-methylxanthine (IBMX) (all from Sigma-Aldrich), 10% FBS, and PenStrep in HG-DMEM. We noticed that adipogenic induction was not successful using conventional adipogenic induction medium, which appeared to be toxic. We then tested whether a single ingredient could be removed from the adipogenic induction medium to eliminate the toxicity, and if this modified medium would support oBMSC adipogenic induction. We included a hBMSC population in this adipogenic assay for comparison.

To assess osteogenic and adipogenic induction, monolayers were fixed for 20 min in 4% paraformaldehyde (PFA), then washed and stained. Mineralised matrix deposition was assessed in osteogenic cultures using Alizarin Red S staining (Sigma-Aldrich). Lipid vacuoles in adipogenic cultures were stained using Oil Red O (Sigma-Aldrich). Wells were rinsed with distilled water, incubated with Alizarin Red S or Oil Red O stain for 10 min, then washed with water and visualised.

### Chondrogenic induction cultures

To induce chondrogenesis, cells were resuspended in chondrogenic medium composed of HG-DMEM, PenStrep, GlutaMax, 1X ITS-X, 100 μM sodium pyruvate (all from Thermo Fisher Scientific), 10 ng/mL TGF-β1 (PeproTech), 100 nM Dex, 200 μM ascorbic acid 2-phosphate, and 40 μg/mL l-proline (Sigma-Aldrich). Prior to cell seeding, 3 mL of cell-free chondrogenic induction medium was added to each well, and plates were centrifuged for 5 min at 2000×*g* to ensure elimination of air bubbles from microwells in the Microwell-mesh. To generate micro-pellets, each well was seeded with 1.2 × 10^6^ oBMSC in 1 mL of chondrogenic medium and the plate was centrifuged at 500×*g* for 3 min to force the cells through the nylon mesh and into the microwells. The microwell array had ~ 250 microwells per well, and therefore, ~ 5000 cells were seeded per micro-pellet. At culture harvest, the nylon mesh was peeled off of the PDMS microwell insert to liberate micro-pellets. Control macro-pellet cultures were established by seeding 2 × 10^5^ oBMSC in 1 mL of induction medium in 96-well, deep V-bottom plates (Corning). Cultures were maintained at 2%, 5%, or 20% O_2_, and 5% CO_2_ in a 37 °C incubator for optimisation experiments, where indicated. Media was exchanged every second day.

### Quantification of glycosaminoglycans (GAG) and DNA

Tissues were incubated in an overnight papain digest (1.6 U/mL papain, 10 mM l-cysteine; both from Sigma-Aldrich) in a 60 °C water bath. The 1,9-dimethylmethylene blue (DMMB, Sigma-Aldrich) assay was used to quantify GAG in the digested tissues [8]. Chondroitin sulfate from shark cartilage (Sigma-Aldrich) was used to generate a standard curve. DNA content in the tissues was quantified using the Quant-iT™ PicoGreen® dsDNA assay kit (Thermo Fisher Scientific) as per the manufacturer’s protocol.

### Quantitative PCR (qPCR)

At harvest, tissues were collected and stored in 350 μL RLT buffer (Qiagen) containing β-mercaptoethanol (Sigma-Aldrich) at − 80 °C. The samples were crushed in 1.5-mL microcentrifuge tubes using sterile micropestles; then, RNA was isolated using the RNeasy Mini Kit (Qiagen) with on-column DNase I (Qiagen) digestion, as per the manufacturer’s instructions. RNA was quantified with a NanoDrop Lite spectrophotometer (Thermo Fisher Scientific), and RNA was reverse-transcribed using the SuperScript III First-Strand Synthesis System for RT-PCR (Thermo Fisher Scientific) as described by the manufacturer. The master mix included 2X SYBR Green PCR Master Mix (Applied Biosystems), 200 nM of the forward and reverse primers, RNase-free water, and 1 μl of sample cDNA. The 5 μl reactions were run in triplicate in a 384-well plate inside a Viia7 Real Time PCR System (Applied Biosystems). The initial cycle was 50 °C for 2 min and 95 °C for 10 min, followed by 40 cycles of 95 °C for 15 s and 60 °C for 1 min. The melt curve and electrophoretic gels were evaluated to confirm the specificity of products. Primer set information for genes of interest is provided in Table 1. Gene expression values were normalised to GAPDH and calculated using the ΔCt method.

**Table 1:** Primers used for qPCR for ovine genes

Gene	Forward	Reverse	Amplicon (bp)	Accession #
*ACAN*	TTTGGACTTTGGCAGAATACC	AATCCAGAAGGAAGACCACTTG	78	FJ200438.1
*COL2A1*	CTGTCCTTCGGTGTCACGG	CGGGCTTCCACACATCCTTAT	93	XM_027967399.1
*COL1A1*	CAGGGAGACAGAGGCATCAAG	ATCTTTGCCAGGAGTACCAGC	156	XM_027974706.1
*COL10A1*	GCATAAAAGGCCCACCATCC	CTGGTGGACCGGGGATAC	88	XM_004011185.4
*SOX9*	CAAGCTCTGGAGACTGCTGAA	CCGTTCTTCACCGACTTCCT	135	XM_027974011.1
*RUNX2*	CGCCTCACAAACAACCACAG	GCTTGCAGCCTTAAATGACTCT	143	XM_027959124.1
*GAPDH*	ACAGTCAAGGCAGAGAACGG	CCAGCATCACCCCACTTGAT	98	NM_001190390.1

### Histology and immunohistochemistry

Tissues were washed in PBS and fixed in 4% PFA for 20 min and frozen in Tissue-Tek OCT compound (Sakura Finetek). Samples were cryosectioned at 7 μm and collected onto poly-lysine coated slides and then frozen until further processing. The sectioned tissues were fixed for 15 min with 4% PFA and washed with PBS. Sections were stained with Alcian blue (Sigma-Aldrich) for 30 min to visualise GAG distribution. Slides were rinsed with tap water and sections were counterstained with Nuclear Fast Red for 5 min, rinsed with tap water, and mounted and coverslipped. For immunohistochemical staining, sections were treated with hyaluronidase (2 U/mL, Sigma-Aldrich) for 30 min at 37 °C. Slides were washed twice with 0.025% Triton X-100/PBS, then blocked for 60 min at room temperature with 10% normal goat serum (ThermoFisher Scientific). Primary antibodies (all from Abcam) raised against collagen type I (1:800; ab6308), type II (1:100; ab34712), and type X (1:100; ab58632) were diluted in 1% BSA/PBS and incubated on sections at 4 °C, overnight. Sections were rinsed twice for 5 min with 0.025% Triton X-100/PBS. Slides were incubated with 0.3% H_2_O_2_ for 15 min and rinsed twice with PBS. Sections were incubated for 60 min at room temperature with secondary antibodies, goat anti-rabbit (HRP; ab6721) or goat anti-mouse (HRP; ab97023), at 1:1000 (Abcam) in 1% bovine serum albumin (BSA)/PBS, then washed twice with PBS. A DAB chromogen kit (Abcam) was used to develop the signal for 8 min; then, slides were rinsed in water, mounted, and coverslipped.

### Autologous repair of in vivo cartilage defects in sheep

We evaluated oBMSC micro-pellets and oACh micro-pellets in vivo, in sheep cartilage defects. Due to the high cost of sheep and the complexity of the procedure, we performed a pilot study with 2 sheep for oBMSC and 2 sheep for oACh micro-pellet evaluation. The procedure was carried out in two steps. In a first procedure, bone marrow aspirates or articular cartilage were collected from 2 sheep each (total of 4 sheep). The cells were isolated, expanded, and frozen at P0, as described above. In a second procedure, cells were thawed, expanded for one additional passage, induced for 10 days in Microwell-mesh cultures, and implanted in cartilage defects created in the trochlear groove of knees of the same sheep from which the original cells were sourced (autologous implantation). All cell culture was carried out in an incubator set at 2% O_2_, 5% CO_2_, and 37 °C. Sheep that received oACh micro-pellets received the implanted tissue in the knee opposite to the one where the original cartilage biopsy had been collected. Some micro-pellets were saved for GAG staining and qPCR analysis. Sheep were anaesthetised and prepared for surgery. Following exposure of the femoral trochlear groove, three 6-mm defects were made in the trochlear groove using a 6-mm biopsy punch, and the articular and calcified cartilage was removed using a curette until the subchondral bone was exposed (~ 1.5 mm deep). Approximately 5 mm of intact cartilage was left between defects. The defect sites and exposed cartilage were irrigated with saline regularly to clean and maintain tissue moisture.

To prepare the cartilage constructs, we used custom well chambers that consisted of a glass slide and a cylindric well made from a PDMS ring that had an internal diameter of 6 mm and 1.5 mm depth. The well chambers were sterilised by autoclaving and handled aseptically. A 6-mm wide disc was punched from a sheet of CelGro™ (Orthocell Ltd., Perth, Australia) collagen scaffold and placed in the bottom of the well. A dropper was used to drop micro-pellets into the well on top of the CelGro scaffold. Excess liquid was removed from the chamber with a pipette tip. Once the well was adequately filled with micro-pellets, two drops of fibrinogen were applied, followed by two drops of thrombin, both from a Baxter Tisseel fibrin glue kit. The gelled construct was removed from the chamber using forceps and inserted into the cartilage defect with the micro-pellets facing the subchondral bone and the CelGro scaffold sitting on top, adjacent to the superficial cartilage layer. To provide a scaffold-only control, one defect site in both the oBMSC and oACh sheep groups was filled with fibrin glue and the Celgro scaffold, but no cells. Four degradable sutures were used to fix the membrane to adjacent cartilage tissue. The patella was returned to its natural position and the incision was sutured to close the knee joint. Knee joints were treated with antibiotic and bandaged for the oACh animals, and a plaster cast was additionally applied on the oBMSC animals. Plaster casts were introduced in the oBMSC group, in an effort to further reduce leaning on the operated joint, which was observed with the first group of animals (oACh group). Both groups of sheep recovered in slings for 2 weeks in an effort to limit the weight on the repaired joint and then were permitted to roam freely for the remaining 6 weeks. A total of 8 weeks after the procedure, sheep were euthanised and joints recovered. The defect regions were trimmed with a saw and fixed in 4% PFA for 72 h. The defects were decalcified using a KOS rapid decalcification machine (Milestone). The tissues were dehydrated, paraffin embedded, and sectioned at 5 μm. The tissues were stained with haematoxylin and eosin (H&E), as well as Alcian blue and Nuclear Fast Red.

### Statistical analysis

Statistical significance of quantitative data was performed using ANOVA in GraphPad Prism. Statistical significance was set at *P* < 0.05. For GAG, DNA, and qPCR analysis, each culture was performed for 4 replicate wells (*n* = 4). The number of unique cell donors or animals was as indicated in the “Materials and methods” and “Results” sections.

## Results

### oBMSC characterisation

**Fig. 1:**
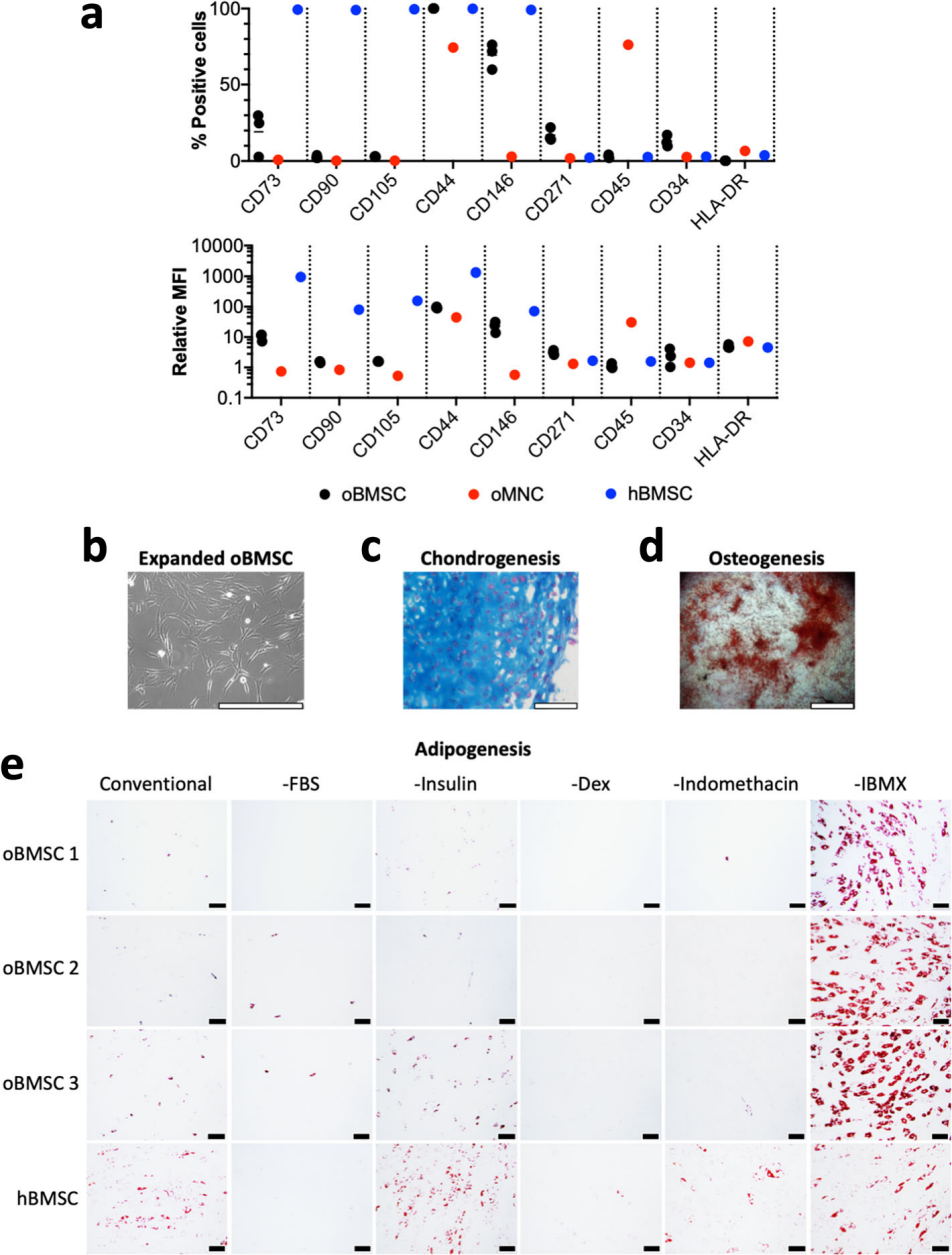
oBMSC characterisation. **a** Flow cytometry analysis of three unique oBMSC donors (black circles), with ovine MNC (oMNC, red circles) and hBMSC (blue circles) as control populations. The upper graph shows the percentage of cells that were positive for the markers. The lower graph shows the mean fluorescence intensity (MFI) of the markers for each cell population. Flow cytometry histograms for each donor and each marker are shown in Supplementary Figures 1, 2, 3, 4 and 5. **b** Bright-field image of oBMSC during expansion. **c** Chondrogenic induction shown by Alcian blue staining of matrix GAG. **d** Osteogenic induction of oBMSC showing mineralised nodules stained with Alizarin Red S. **e** Adipogenic induction of three unique oBMSC donors and an hBMSC donor, showing that conventional induction media does not result in adipogenesis of oBMSC unless IBMX is removed. Lipid vacuoles are stained red with Oil Red O. Scale bar = 400 μm in **b** and **c**, 2 mm in **d**, and 100 μm in **e**

Isolated oBMSC appeared spindle-shaped upon expansion (Fig. 1b), resembling BMSC from other species such as hBMSC. Flow cytometry analysis is summarised in Fig. 1a for oBMSC populations derived from three unique donors, one sheep bone marrow mononuclear cell (MNC) donor population, and one hBMSC donor population (histograms are shown in Supplementary Figures 1, 2, 3, 4 and 5). The Fig. 1a upper graph displays the percentage of cells that expressed the antigen, while the lower graph displays the relative mean fluorescence intensity (MFI) of each antibody on each cell population. Unlike hBMSC, oBMSC contained only a small population of cells positive for CD73 (10.2% ± 12.7%), were negative for CD90 and CD105, and contained a small population positive for CD34 (13.1% ± 3.7%). Like hBMSC, oBMSC were 100% positive for CD44, mostly positive for CD146 (69% ± 8.4%), and had a small population that was positive for CD271 (17.0% ± 4.3%). The low frequency of CD45^+^ cells (3.1% ± 1.1%) in oBMSC cultures, along with data indicating that 76% of sheep MNCs stained positively for CD45, suggests that the CD45 antibody works, and that the expanded oBMSC had been depleted of CD45^+^ cells. Given the successful depletion of CD45^+^ cells during oBMSC enrichment and expansion, the small population of CD34^+^ cells (13.1% ± 3.7%) likely reflects non-specific binding with this antibody. While the antibody panel had limited cross-reactivity with oBMSC, the relative enrichment of CD146^+^ cells [17] and depletion of CD45^+^ cells [18] suggested that these cells were suitable for use in further characterisation studies.

The multipotency of oBMSC was evaluated using in vitro tri-lineage differentiation assays, following protocols conventionally used for hBMSC. Alcian blue staining of oBMSC macro-pellets cultured in chondrogenic induction medium demonstrated deposition of cartilage-like extracellular matrix (ECM; Fig. 1c). Alizarin Red S staining confirmed calcium nodules in osteogenically induced cultures (Fig. 1d). Following adipogenic induction using conventional induction medium, we noticed that adipogenesis was poor and the cells appeared unhealthy and detached from the well plates. To test whether there was a component in the conventional adipogenic medium that was toxic to oBMSC, we set up an experiment in which we removed one component at a time from the conventional adipogenic medium. We also included an hBMSC population in this test as a control. We found that removal of IBMX resulted in healthy oBMSC cultures that underwent adipogenesis, forming ample lipid vacuoles, demonstrated by Oil Red O staining (Fig. 1e). Removal of IBMX from hBMSC adipogenic induction cultures did not demonstrate the same stark difference, relative to conventional adipogenic induction medium.

### Growth of macro-pellets and micro-pellets in the microwell-mesh system

**Fig. 2:**
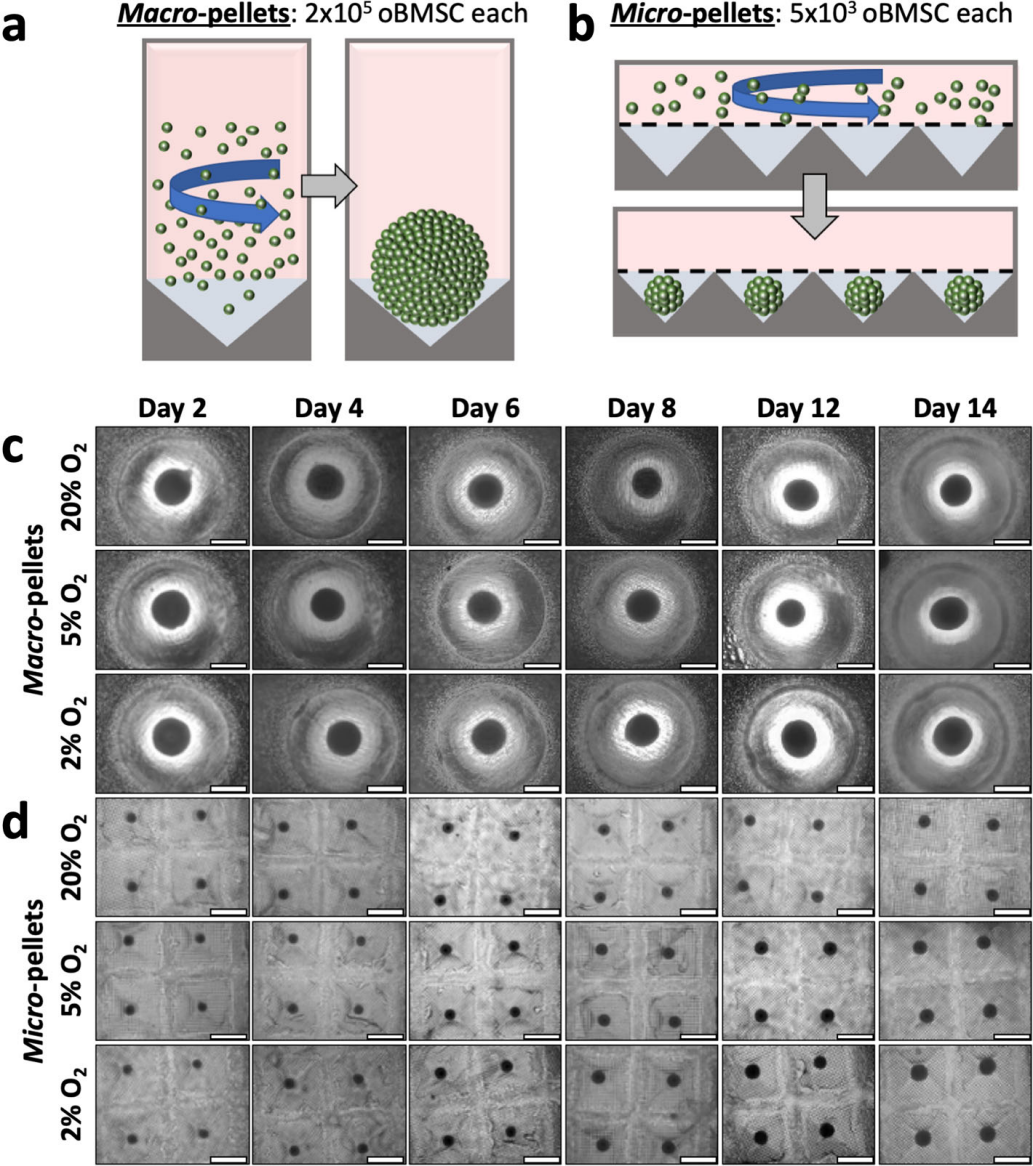
Schematic of macro-pellet and micro-pellet assembly. **a** Macro-pellets were assembled by centrifuging 2 × 10^5^ oBMSC in V-bottom deep well plates. **b** Micro-pellets were assembled by centrifuging 5 × 10^3^ oBMSC per microwell, in the Microwell-mesh (**a** and **b** adapted from [7]). Microscopic images over a 14-day culture period of **c** macro-pellets cultured in deep-well plates and **d** micro-pellets cultured in the Microwell-mesh platform. Scale bar = 1 mm. Images for 2 additional oBMSC donors are shown in Supplementary Figure 6

**Fig. 3:**
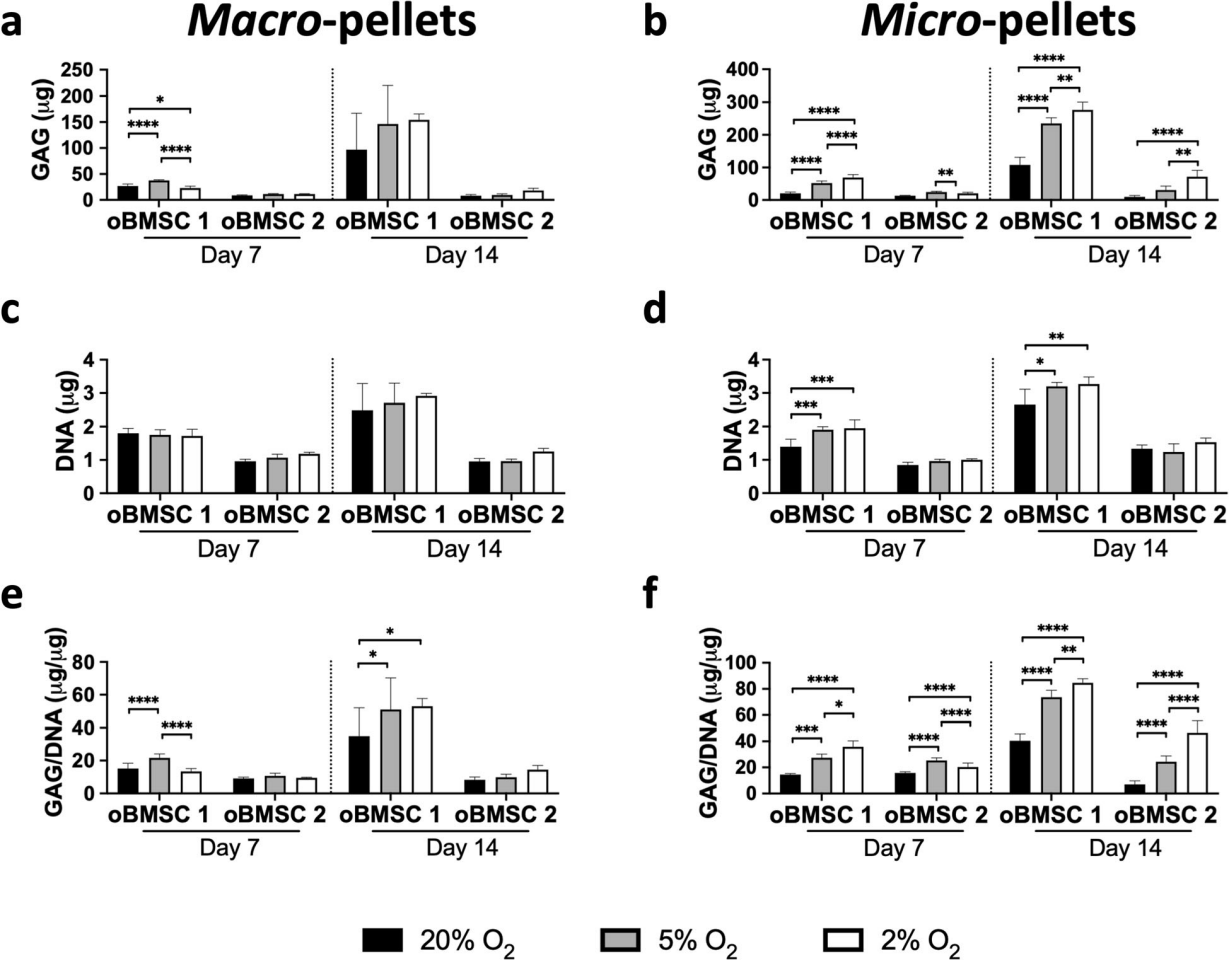
Quantification of GAG and DNA in macro-pellets and micro-pellets on day 7 and day 14 for oBMSC 1 and oBMSC 2. Quantities of GAG in **a** macro-pellets and **b** micro-pellets. Quantities of DNA in **c** macro-pellets and **d** micro-pellets. GAG normalised to DNA in **e** macro-pellets and **f** micro-pellets (*P < 0.05, **P < 0.01, ***P < 0.001, and ****P < 0.0001)

To characterise the chondrogenic potential of oBMSC, we assessed chondrogenesis in both our custom Microwell-mesh culture platform (5 × 10^3^ cells/micro-pellet, Fig. 2b), as well as traditional macro-pellet culture (2 × 10^5^ cells/macro-pellet, Fig. 2a). Over 14 days of chondrogenic induction culture, oBMSC micro-pellets and macro-pellets in 2%, 5%, and 20% O_2_ atmospheres showed an increase in size (Fig. 2c). Micro-pellets cultured at 2% O_2_ were larger than micro-pellets cultured at 5% O_2_ or 20% O_2_, and this was consistent across 3 unique oBMSC donors (see Supplementary Figure 6 for two additional donors). Some variability was observed across different oBMSC donors, particularly in macro-pellet cultures (compare Fig. 3d with Supplementary Figure 6A and C). However, this is not surprising as donor variability has been well-documented with hBMSC [19].

### Low oxygen increases GAG production in micro-pellets

GAG production increased from day 7 to day 14 for both macro-pellets and micro-pellets, for both of the two unique oBMSC donors analysed, oBMSC 1 and oBMSC 2 (Fig. 3a and b). In macro-pellet cultures, the amount of GAG and DNA trended toward a higher level at lower O_2_ concentrations (2% and 5%), but this was not consistently statistically significant due to a large amount of variability between individual macro-pellets (Fig. 3a, c, and e). In micro-pellet cultures, the amount of GAG was greater at lower O_2_ concentrations (2% and 5%) and this was statistically significant for oBMSC 1 on day 7 and day 14, and for oBMSC 2 on day 14 (Fig. 3b). The amount of DNA in micro-pellets was higher for oBMSC 1 at lower O_2_ concentrations (2% and 5%), compared with 20% O_2_, but the magnitude of differences between O_2_ concentrations was generally small (Fig. 3d). When GAG was normalised to DNA for micro-pellet cultures, the amount of GAG/DNA was greater at lower O_2_ concentrations (2% and 5%), compared with 20% O_2_, and this was statistically significant for both oBMSC donors at both time points (Fig. 3f). There was substantial variability observed between the two oBMSC donors, with oBMSC 1 producing substantially more GAG than oBMSC 2 when assessed at both day 7 and day 14.

As previously observed for hBMSC [3, 7], GAG/DNA production in micro-pellet cultures was typically greater than in macro-pellet cultures. At 5% O_2_, GAG/DNA was 1.44 ± 0.11-fold greater (*P* = 0.0039) in oBMSC 1 micro-pellet cultures than macro-pellet cultures on day 14, and for oBMSC 2, GAG/DNA was 2.27 ± 0.42-fold (*P* < 0.0001) greater in micro-pellet cultures than macro-pellet cultures on day 7. At 2% O_2_, GAG/DNA was greater in micro-pellet cultures than in macro-pellet cultures for both donors, and on both days 7 and 14. For oBMSC 1 at 2% O_2_, GAG/DNA was 2.68 ± 0.34-fold (*P* < 0.0001) and 1.60 ± 0.06-fold (*P* < 0.0001) greater in micro-pellet cultures compared with macro-pellet cultures on days 7 and 14, respectively. For oBMSC 2 at 2% O_2_, GAG/DNA was 4.90 ± 0.96-fold (*P* < 0.0001) greater on day 7 and 3.22 ± 0.63-fold (*P* < 0.0001) greater on day 14 in micro-pellet cultures compared with macro-pellet cultures.

### ECM characterisation in macro-pellets and micro-pellets

**Fig. 4:**
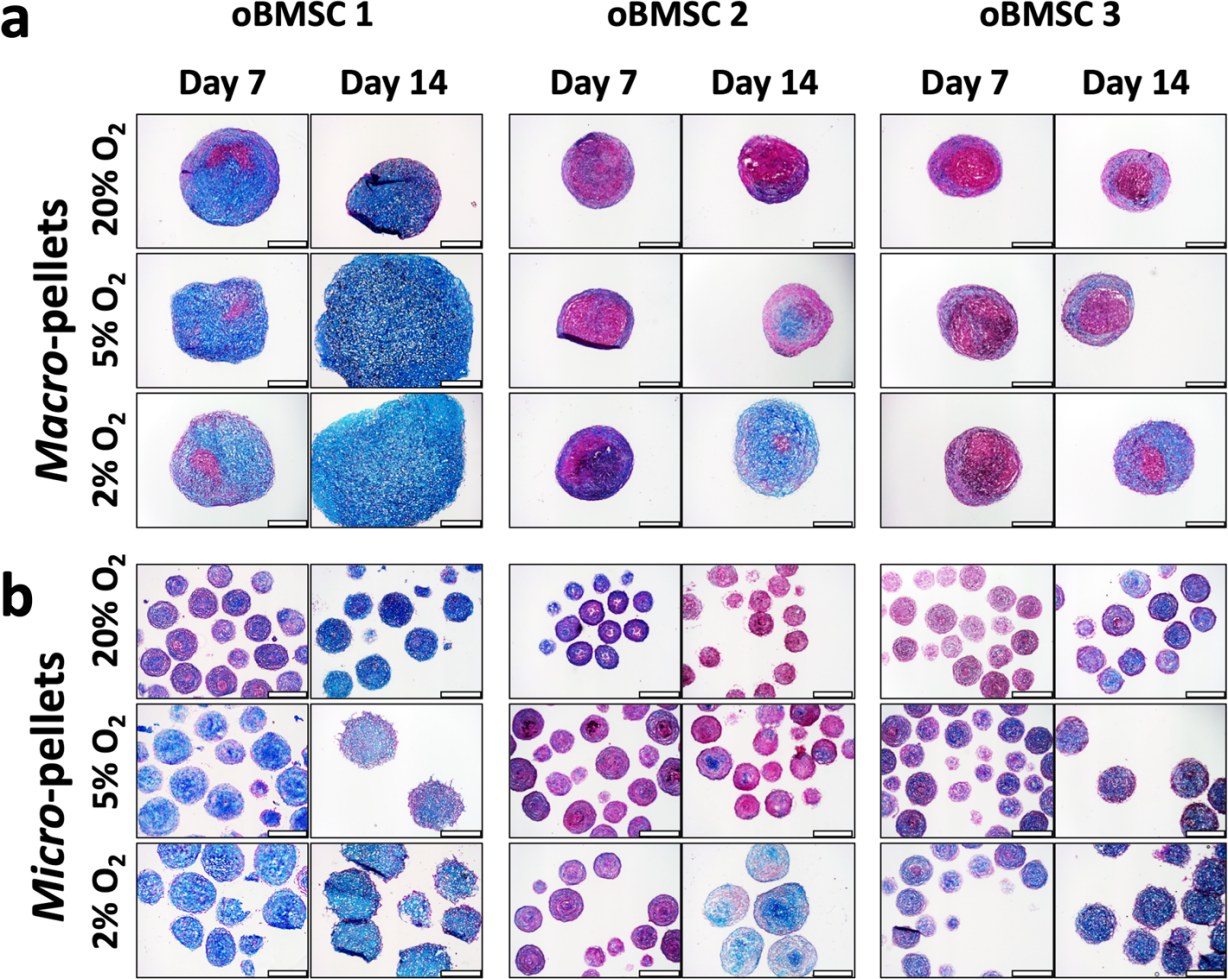
Alcian blue staining for GAG in **a** macro-pellet and **b** micro-pellet sections. Nuclei are stained red. Scale bar = 400 μm

We assessed the production of cartilage-like ECM with 3 unique oBMSC donors in macro-pellets and micro-pellets cultured in 2%, 5%, and 20% O_2_ atmospheres, on day 7 and day 14. Alcian blue staining revealed increased GAG matrix accumulation in both macro-pellets and micro-pellets cultured at 2% O_2_ compared with 20% O_2_ (Fig. 4a and b). On day 7, macro-pellets contained cell-dense cores (red stained nuclei) with little cartilage-like matrix at all oxygen levels (Fig. 4a). For this reason, the Nuclear Fast Red staining is prominent at these early time points. By day 14, macro-pellets exhibited more uniform GAG matrix throughout their diameter for oBMSC 1 at all oxygen concentrations, and for BMSC 2 and BMSC 3 at 2% O_2_. Similarly, micro-pellets cultured at 2% O_2_ stained more uniformly with Alcian blue throughout their diameter by day 14, compared with 5% and 20% O_2_ (Fig. 4b). Consistent with GAG quantification (Fig. 3), variability was also evident in Alcian blue GAG staining between unique oBMSC donors.

We also performed immunohistochemistry staining for cartilage-associated ECM molecule, type II collagen (Supplementary Figure 7); bone-associated molecule, type I collagen (Supplementary Figure 8); and hypertrophy-associated molecule, type X collagen (Supplementary Figure 9). While we observed some staining for all of these collagen molecules, we did not observe a staining pattern that correlated with differing oxygen concentrations.

### In vivo cartilage defects in sheep

**Fig. 5:**
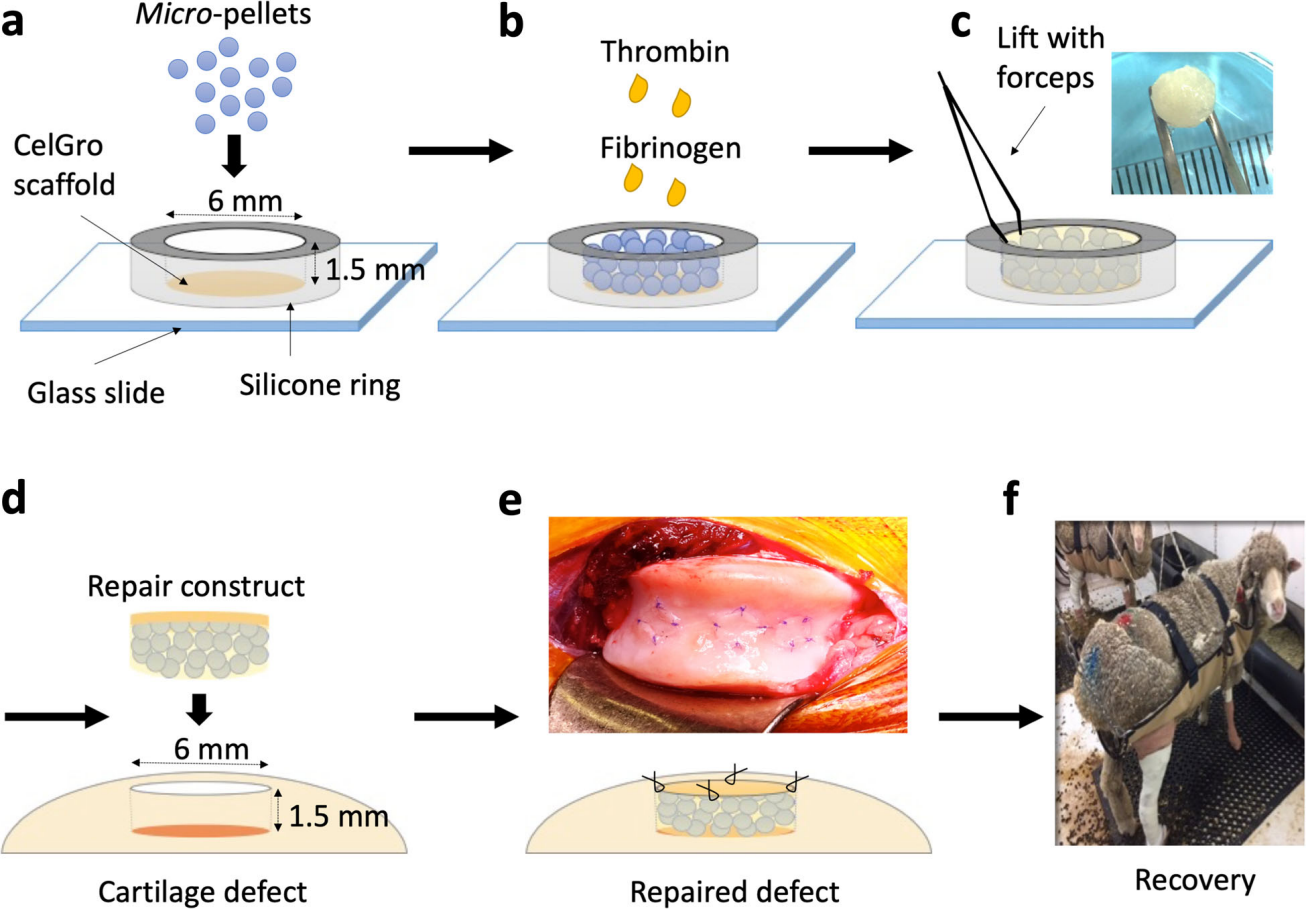
Schematic of in vivo evaluation of micro-pellets. **a** Ten-day chondrogenically induced oBMSC or oACh micro-pellets were layered onto a Celgro scaffold in a custom-made cylindric chamber (6 mm in diameter and 1.5 mm high). **b** Two drops of fibrinogen were added to the micro-pellets, followed by two drops of thrombin, to adhere the micro-pellets to each other and to the Celgro scaffold. **c** The repair construct was lifted with forceps. **d** The repair construct was placed into a cartilage defect that was of a similar dimension, with the micro-pellet layer facing down into the subchondral layer and the Celgro scaffold on top. **e** Four sutures were used to fix the Celgro scaffold to the adjacent cartilage tissue. **f** Sheep were placed in slings for 2 weeks to recover and then were allowed to roam freely for the remaining 6 weeks

We evaluated the potential of oBMSC micro-pellets to repair cartilage defects in sheep. Micro-pellets induced for 10 days were suspended in fibrin glue and anchored to a collagen Celgro scaffold/membrane that was used to keep the tissues fixed in osteochondral defects in sheep (see schematic in Fig. 5). Three defects (6 mm in diameter and 1.5 mm deep) were made in the trochlear groove of one hind leg for each sheep and filled with repair tissues. Two sheep were implanted with micro-pellets made from induced oBMSC, and two sheep were implanted with micro-pellets made from induced oACh, as controls. Sheep were euthanised after a total of 8 weeks from the day of receiving an implant, and defect repair was assessed by histology.

### Histological and qPCR assessment of oBMSC and oACh micro-pellets pre-implant

**Fig. 6:**
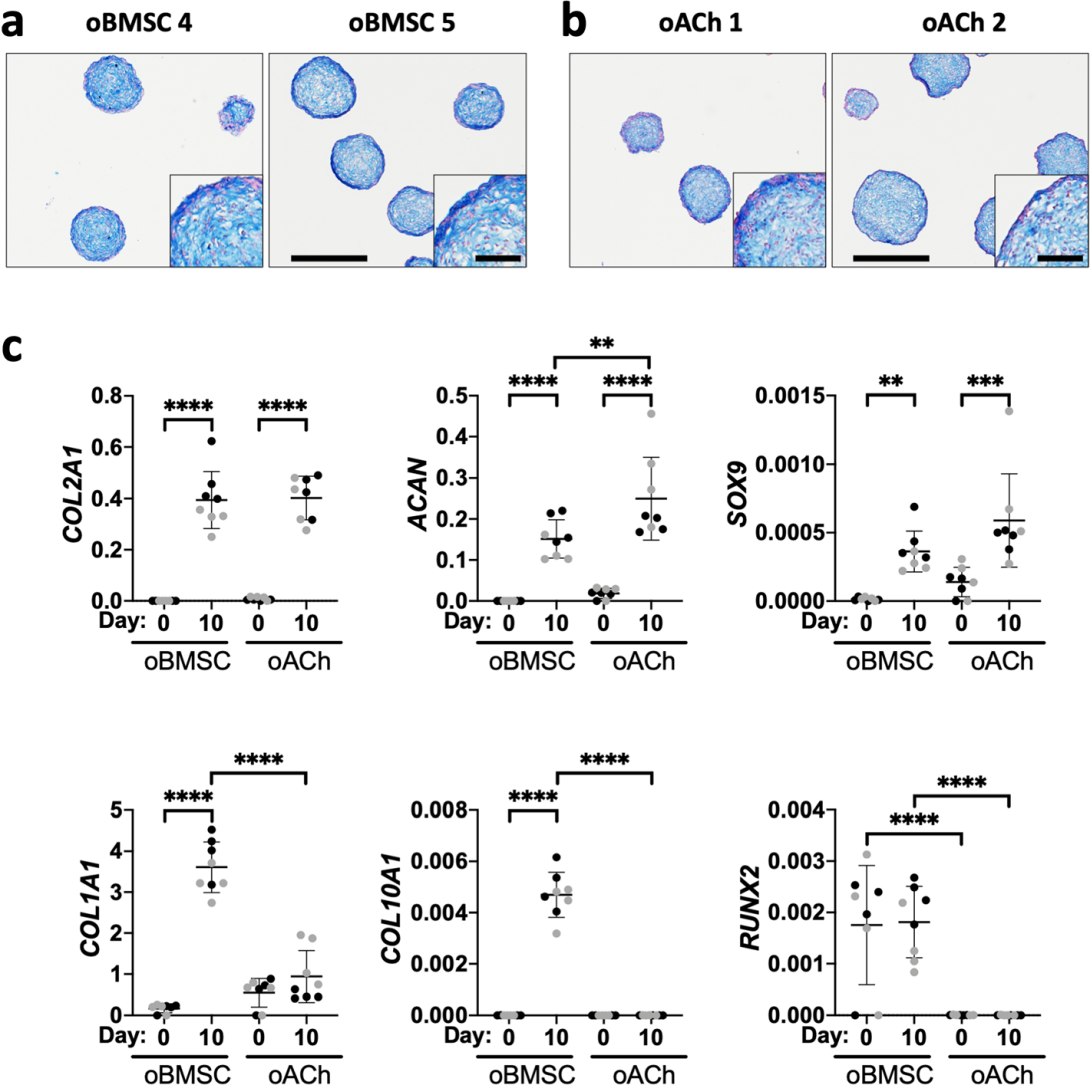
Characterisation of induced micro-pellets used for sheep studies. **a** oBMSC and **b** oACh micro-pellet sections stained with Alcian blue for GAG following 10 days of chondrogenic induction (scale bar = 500 μm and inset scale bar = 100 μm). **c** qPCR analysis of cartilage-like tissues formed from oBMSC and oACh after expansion (day 0) and 10 days after chondrogenic induction (day 10). Relative gene expression was assessed for chondrogenic genes *COL2A1*, *ACAN*, and *SOX9*, and for hypertrophic genes *COL1A1*, *COL10A1*, and *RUNX2*. Gene expression levels were normalised to *GAPDH* and represent 2^-ΔCt values. The two unique donors are distinguished by black versus grey symbols. The mean is represented by the horizontal line (*P < 0.05, **P < 0.01, ***P < 0.001, and ****P < 0.0001)

We assessed the micro-pellets implanted in sheep histologically and for relative gene expression (Fig. 6). Ten-day, chondrogenically induced micro-pellets derived from both oBMSC (Fig. 6a) and oACh (Fig. 6b) donors stained for GAG with Alcian blue, demonstrating the development of cartilage-like tissue. Histologically, the oBMSC- and oACh-derived micro-pellets were visually indistinguishable from each other. qPCR analysis was performed to quantify the relative gene expression (Fig. 6c) for cartilage-associated genes (*COL2A1*, *ACAN*, and *SOX9*) and for bone or hypertrophy-associated genes (*COL1A1, COL10A1*, and *RUNX2*) in both oBMSC- and oACh-derived micro-pellets, at day 0, before induction, and on day 10 of induction. For both oBMSC- and oACh-derived micro-pellets, the expression of *COL2A1*, *ACAN*, and *SOX9* were statistically significantly increased on day 10 following chondrogenic induction, relative to day 0. For oBMSC-derived tissues, *COL2A1*, *ACAN*, and *SOX9* increased by 1.45 × 10^6^ ± 3.82 × 10^5^-fold, 2.56 × 10^4^ ± 7.38 × 10^3^-fold, and 61.3 ± 23.6-fold, respectively, on day 10 relative to day 0. For oACh-derived tissues, *COL2A1*, *ACAN*, and *SOX9* increased by 53.8 ± 10.7-fold, 13.5 ± 5.11-fold, and 4.23 ± 2.29-fold, respectively, on day 10 relative to day 0. *ACAN* expression was 1.65 ± 0.624 higher in oACh micro-pellets following induction (day 10) relative to oBMSC micro-pellets (day 10), whereas *COL2A1* and *SOX9* were statistically similar between oBMSC and oACh at both time points. For oBMSC-derived tissues, the expression of *COL1A1* (21.3 ± 3.41-fold) and *COL10A1* (1.61 × 10^3^ ± 283-fold) were increased following chondrogenic induction, while *RUNX2* expression was not changed following induction. Following induction (day 10), *COL1A1* (8.97 ± 1.44-fold) and *COL10A1* (1.10 × 10^3^ ± 193-fold) were higher in oBMSC-derived micro-pellets relative to oACh-derived micro-pellets. *RUNX2* expression was higher in oBMSC-derived micro-pellets compared with oACh micro-pellets, both on day 0 (345 ± 213-fold) and on day 10 (708 ± 254-fold).

### Histological assessment of oBMSC and oACh micro-pellets post-implant

**Fig. 7:**
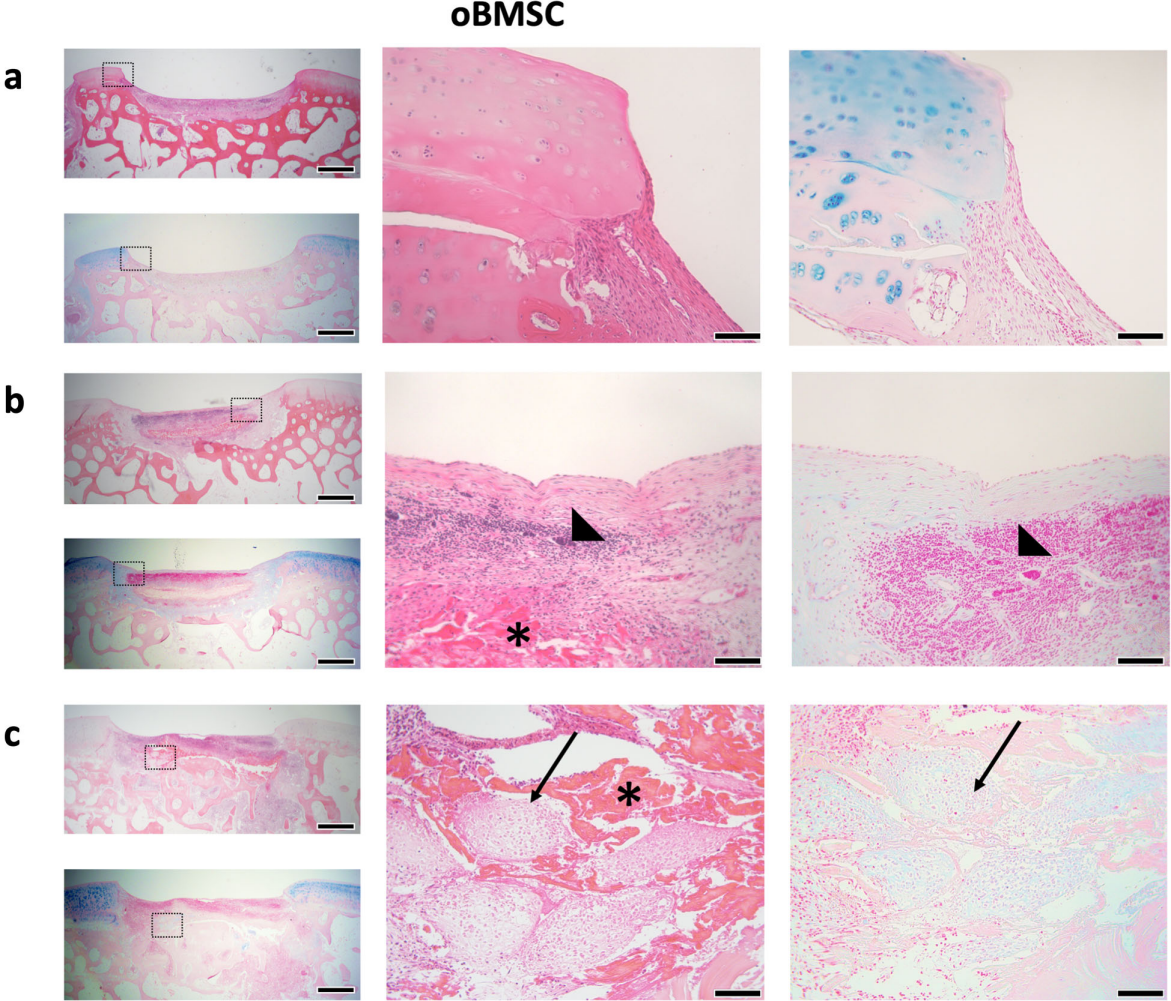
Representative H&E and Alcian blue stained images of in vivo oBMSC micro-pellet repair tissues after 8 weeks. Repair tissue was largely composed of **a** fibrous tissue and **b** fibrous tissue with significant vascular and blood cell infiltration (triangle). **c** In one of 6 defects, a few micro-pellets could be identified; the boxed regions are enlarged to the right, with arrows pointing to micro-pellets. The scaffold was largely degraded, but could be seen in some defects (see asterisks in **b** and **c**). Boxes with dashed lines in images on the left are enlarged in the middle (H&E) and right (Alcian blue) images. Scale bars in left panels = 1 mm. Scale bars in middle and right panels = 100 μm

**Fig. 8:**
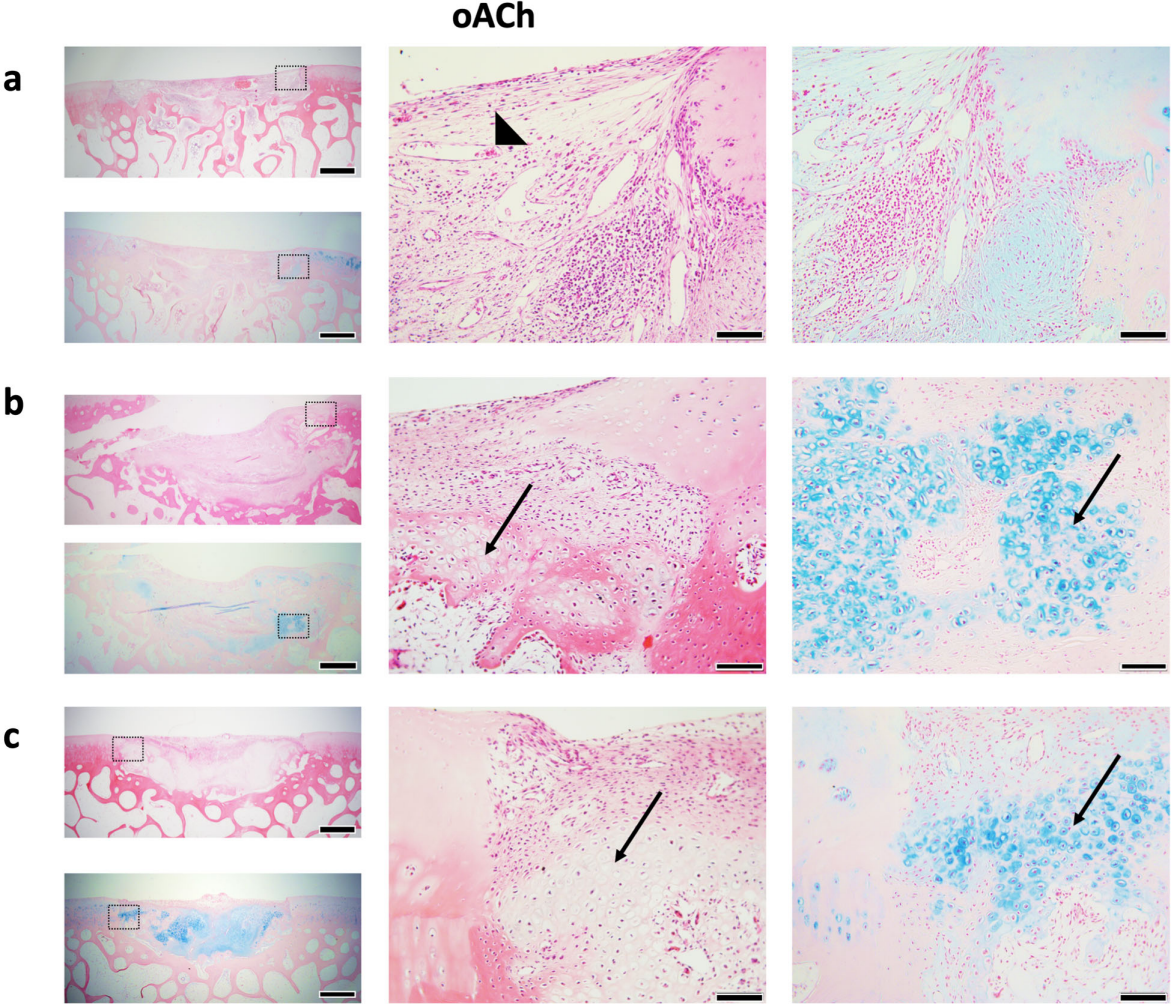
Representative H&E and Alcian blue-stained images of in vivo oACh micro-pellet repair tissues after 8 weeks. Repair tissue was largely composed of **a** fibrous tissue with some vascular tissue (triangle), with **b** one defect showing modest regions of GAG-rich repair cartilage (arrows), and **c** another defect showing more substantial regions of GAG-rich repair cartilage (arrows). Boxes with dashed lines in images on the left are enlarged in the middle (H&E) and right (Alcian blue) images. Scale bars in left panels = 1 mm. Scale bars in middle and right panels = 100 μm

Following 8 weeks of incubation in vivo in sheep, joints were harvested, and defect repair was characterised histologically (Figs. 7 and 8). None of the oBMSC or oACh micro-pellet groups showed complete cartilage fill or seamless integration with adjacent tissue. Figure 7 shows three representative defects that were filled with oBMSC-derived micro-pellets and stained with H&E and Alcian blue. H&E staining showed that most of the defects were filled with fibrous tissue, vascular tissue, and blood cells (Fig. 7a–c). There was faint Alcian blue staining for GAG in some of the defects (see Fig. 7b, lower left panel), suggesting either residual cartilage from the implanted micro-pellets, or that cartilage repair was occurring very slowly. This could also have been from infiltration of chondroprogenitors from adjacent tissue rather than oBMSC. Supplementary Figure 10 shows histology of a scaffold-only control, where the defect was filled with fibrin glue and the Celgro scaffold, but no cells. Of the 5 defects filled with oBMSC micro-pellets, we could only find one defect that still had some recognisable micro-pellets, which are shown Fig. 7c. Based on the morphology of these oBMSC micro-pellets, it appeared as though they may have been in the process of dedifferentiating or being absorbed by surrounding tissue.

Figure 8 shows three representative defects that were filled with oACh-derived micro-pellets and stained with H&E and Alcian blue. H&E staining showed that most of the defect was filled with fibrous tissue, but unlike defects filled with oBMSC micro-pellets, they generally appeared to have attracted fewer blood lineage cells and less vascular tissue. Of the 5 defects filled with oACh micro-pellets, we could only find two defects that still had substantial cartilage-like tissue fill, which are shown in Fig. 8b and c. There was still a significant amount of fibrous tissue observed in these defects; however, they showed the most promising result in terms of the amount of Alcian blue-stained GAG matrix and morphology of the cells, which appeared to form lacunae. Supplementary Figure 10 shows histology from an empty Scaffold-only control defect site. While oACh-derived micro-pellets yielded more promising histology, macroscopic imaging of the joint revealed that defects remained visible, with substantial repair visible only in a single defect (Supplementary Figure 11).

## Discussion

In this study, we characterised oBMSC expansion cultures in vitro, used a micro-pellet model to identify the optimal oxygen atmosphere for oBMSC chondrogenesis, and then compared the capacity of micro-pellets assembled from either oBMSC or oACh to repair osteochondral defects in adult sheep.

oBMSC were successfully isolated using plastic adhesion and cells exhibited a spindle-shaped morphology similar to hBMSC. Expanded oBMSC were characterised using a flow cytometry antibody panel commonly used to characterise hBMSC [20, 21], including CD73, CD90, CD105, CD44, CD146, CD271, CD45, CD34, and HLA-DR. Like our control hBMSC population, expanded oBMSC were essentially negative for haematopoietic markers (CD45 and CD34), strongly positive (~100%) for CD44, contained a large population (~ 70%) of CD146^+^ cells, and had a very small population (< 3%) of CD271^+^ cells. CD146 is viewed as one of the defining markers of hBMSC and is associated with hBMSC capacity to form ectopic marrows and support haematopoietic stem cells [22, 23]. CD271 has been reported to be expressed on the surface of hBMSC [24] and oBMSC [25]. However, CD271^+^ cell number in hBMSC cultures declines rapidly in response to medium serum supplementation [26], and our hBMSC control population and oBMSC cultures also had few CD271^+^ cells. We did not observe positive staining of oBMSC using anti-human CD90 or CD105 antibodies (< 3%), and only two oBMSC donors stained weakly for CD73 (< 30%), while one donor was essentially negative for CD73 (< 3%). A previous study also reported poor cross-reactivity of oBMSC surface antigens and anti-human CD73, CD90, and CD105 antibodies [27]. As expected, oBMSC were negative for the human-specific marker, HLA-DR, which served as a negative control. Other markers that have been positively detected on oBMSC include CD166 and CD29 [28]. While the full panel of hBMSC antibody markers did not react against oBMSC, we were able to confirm the effective depletion of haematopoietic cells, and that the plastic-enriched cells displayed some overlap with known hBMSC antigens, which can be used as putative oBMSC markers in future studies.

Using conventional tri-lineage induction media, oBMSC underwent osteogenic and chondrogenic induction successfully, while initially, adipogenic induction failed. Using conventional adipogenic induction medium, we observed substantial cell death. By removing one component of the induction medium at a time, we identified that IBMX was toxic to oBMSC. Exclusion of IBMX from the conventional adipogenic induction medium resulted in robust adipogenesis of oBMSC. IBMX is a competitive non-selective phosphodiesterase inhibitor [29], commonly included in BMSC adipogenic induction medium [30]. While other studies do not specifically report the toxicity of IBMX during oBMSC adipogenesis, a previous publication showed an image of a single cell from hBMSC and oBMSC cultures after 14 days of induction in IBMX-supplemented medium [28, 31]. In these images, the adipogenic induced hBMSC appeared healthy and contained many lipid vacuoles, while the oBMSC appeared relatively small with few lipid vacuoles [31]. Another study induced oBMSC in medium supplemented with IBMX and displayed cells in a relatively sparse monolayer, with few lipid vacuoles [32], potentially indicating some level of toxicity. These outcomes are similar to our own observations where only a few small oBMSC remained in adipogenic cultures supplemented with IBMX. For effective oBMSC adipogenic induction, exclusion of IBMX from induction cultures appears to be necessary. Why oBMSC respond differently to IBMX during adipogenic induction, compared with hBMSC, is not immediately obvious. This toxicity, however, identifies an important difference in the biology of oBMSC and hBMSC and highlights the importance of characterising and comparing cells of different species independently.

We characterised the capacity of oBMSC to form cartilage-like tissue in micro-pellet and traditional macro-pellet cultures, in 2%, 5%, or 20% O_2_ atmospheres. A previous study that included both an oBMSC pellet model and mathematical modelling concluded that optimal oBMSC chondrogenesis occurs at 10–11% O_2_ [33]. Another study demonstrated that superior chondrogenesis could be achieved if the oBMSC were expanded in a 5% O_2_ atmosphere, rather than at 20% O_2_ atmosphere [34]. In this previous study, oBMSC expanded in 5% or 20% O_2_ atmospheres were assembled into macro-pellets for chondrogenic differentiation [34]. In our study, we expanded all oBMSC in a 2% O_2_ atmosphere and observed incrementally greater GAG production when oBMSC were assembled into a micro-pellet model and cultured at 2% O_2_, compared with 5% or 20% O_2_ atmospheres. GAG/DNA output was greatest in micro-pellets cultured in 2% O_2_ atmosphere. In macro-pellet cultures, GAG/DNA content trended toward higher levels in lower O_2_ atmospheres, but this was not consistently statistically significant, and greater variability in data was observed in macro-pellet cultures. At 2% O_2_, GAG/DNA was significantly greater in micro-pellet cultures relative to macro-pellet cultures. This pattern of improved chondrogenesis both in micro-pellets and at 2% O_2_ was previously observed in hBMSC chondrogenic cultures [3]. For both oBMSC (in this study) and hBMSC (previous studies [3, 7]), more homogeneous tissue was generated using micro-pellet cultures, relative to macro-pellet cultures. The effect of atmospheric O_2_ concentration was more discernible in homogeneous micro-pellet models, demonstrating that, as with hBMSC chondrogenesis, oBMSC chondrogenesis appears to be more efficient at lower O_2_ concentrations. Across all oBMSC donors, the greatest GAG/DNA was observed in micro-pellets, compared to macro-pellets, at optimal conditions. These data demonstrate that the per-cell matrix output is maximised in the micro-pellet model.

Next, we sought to evaluate the use of autologous oBMSC micro-pellets in an in vivo sheep pilot study. Since autologous ACh are already being used in various clinical cartilage repair strategies [35], we included expanded oACh micro-pellets for comparison. A 10-day chondrogenic induction culture, rather than 14 days, was selected based on a previous study that indicated that following ~ 10 days of culture, micro-pellets gained significant mass and yet still retained their propensity to amalgamate into a continuous tissue [10]. We characterised oBMSC and oACh micro-pellets from 2 unique donors each, using histology and qPCR. By day 10, both oBMSC and oACh micro-pellets stained for GAG with Alcian blue, and both tissues exhibited similar tissue morphology. Conversely, qPCR analysis revealed that hypertrophic gene expression (*COL10A1* and *RUNX2*) was absent in oACh micro-pellets, but significantly over-represented in oBMSC micro-pellets. Hypertrophic signalling and upregulation of endochondral ossification pathways is a well-characterised problem in hBMSC chondrogenic cultures and is viewed as a major impediment to BMSC-mediated cartilage repair [7, 36]. It is common to demonstrate hBMSC hypertrophy by implanting tissue ectopically in immune compromised mice [3, 7, 22, 37]. However, it is unclear if the highly vascularised ectopic microenvironment exacerbates the propensity of hBMSC to undergo hypertrophy, and if hypertrophy would be less problematic in the less vascular environment found in a synovial joint. For this reason, there is merit in evaluating the capacity of BMSC to regenerate cartilage in actual cartilage defect models.

To assess the potential of micro-pellets to repair osteochondral defects, we performed a large animal pilot study using sheep and micro-pellets formed from autologous cells. Two sheep were assigned to the oBMSC group and 2 sheep were assigned to the oACh group. Three replicate defects were created in one stifle joint on each animal. All defects were 6 mm in diameter and 1.5 mm deep, penetrating through the cartilage and into the subchondral bone. Micro-pellets were packed into cylindrical moulds, bonded to a Celgro™ collagen membrane with fibrin glue, and then anchored into defect sites with fibrin glue and sutures (see Fig. 5). During post-surgical recovery, slings were used to partially reduce weight-bearing on the treated sheep joints and plaster casts were introduced to prevent joint mobility. Our ethics protocol did not allow for complete unweighting of the repaired joint. In clinical cartilage repair procedures such as MACI (reviewed here [38]), human patients are advised to adhere to strict progressive weight-bearing and passive motion following these procedures. As the sheep knee joint is high up on the animal’s flank, it is challenging to immobilise this joint with wrapping or a cast. Despite the formation of three 6-mm diameter osteochondral defects in one stifle joint, animals did not appear to be in pain or appear to reduce load on the repaired leg while in the sling or after release from the sling. Full unloading of the treated joint for 1 week or more could potentially improve engraftment and the stability of repair tissues.

Following 8 weeks of in vivo incubation, defects filled with oBMSC micro-pellets were not effectively repaired. oBMSC micro-pellets were visible in 1 of 5 defect sections, but the lacunae structure and Alcian blue staining suggested that the cartilage-like tissue was either de-differentiating or being absorbed by surrounding tissue. H&E staining suggested that most of the defect fill volume contained fibrous tissue, rather than cartilage-like repair tissue. A layer of tissue was stained faintly with Alcian blue at the base of some defect sites, but it is possible that this tissue was derived from adjacent native tissue, or residual oBMSC micro-pellets, rather than new cartilage tissue. Repair from adjacent tissue might be consistent with the concept that BMSC-based therapies can upregulate endogenous regenerative processes [39]. However, this repair was modest and our limited controls did not allow for a statistical evaluation of this outcome. In previous studies, we implanted hBMSC micro-pellets subcutaneously in NSG mice, and after 8 weeks, we observed mineralisation and the formation of marrow-like structures [3]. This is consistent with a wide body of literature that suggests that chondrogenically induced hBMSC appear to engage intrinsic endochondral ossification programming, triggering vascular penetration, ossification, and support of marrow formation [36, 40]. However, in this study, we observed no evidence of mineralisation or marrow formation from oBMSC micro-pellets implanted into osteochondral defect sites. Another study that implanted oBMSC in TGF-β1-laden gelatin foams in sheep tibial growth plates similarly reported a lack of mineralised tissue, with mostly fibrous tissue present after 5 weeks [41]. It is possible that during the incubation period in our studies, micro-pellet mineralisation occurred, but that this tissue was completely re-absorbed by immune cells. Alternatively, it is possible that the microenvironment within these joint defect sites might mitigate mineralisation of tissue, relative to more vascularised microenvironments such as a subcutaneous mouse pouch. While we did not see evidence of mineralisation at 8 weeks, oBMSC micro-pellets did not yield promising cartilage-like repair tissue.

In the oACh micro-pellet group, evidence of cartilage repair tissue was visible in 2 of 5 histology sections. While there was evidence of continuous cartilage-like tissue formation in these regions, there were also regions of fibrous tissue and vasculature. Where cartilage repair was visible, this repair tissue filled both the articular cartilage defect and the subchondral bone region. Mature lacunae structures and Alcian blue staining were visible in this repair tissue. Longer incubation periods beyond 8 weeks would be required to determine if repair tissue integration and a high-quality cartilage repair tissue might evolve. Many sheep studies are carried out for longer periods (8, 10, and 12 weeks [42]; 3 months [43]; 4–12 months [44]), and we presume that promising aspects of repair with oACh micro-pellets would have improved with time. The low success rate of establishing a repair tissue in the oACh group may also be improved by further reducing weight-bearing and motion of the joints during the first few weeks of recovery.

The success of BMSC in cartilage repair is dependent on the development of strategies that promote the formation of stable articular cartilage and mitigate hypertrophy. In recent work, we characterised hBMSC chondrogenic and hypertrophic differentiation cascades in response to TGF-β1 [7]. We observed that in response to as little as a single day of TGF-β1 exposure, intrinsic hypertrophic signalling in hBMSC is activated. *SP7*, a transcription factor that drives bone formation and hypertrophy [45, 46], was upregulated following this brief TGF-β1 exposure and persisted even when TGF-β1 was withdrawn. Knockdown of *Sp7* in mice reduces hypertrophy [45], and SP7 would be a logical drug or genetic engineering target to prevent hypertrophy. Other logical targets to obstruct hypertrophy include BMP2 and WNT11 pathways, which have been trialled with some success in previous hBMSC studies [47, 48]. Currently, chondrogenic stability and the absence of hypertrophy remain significant obstacles for the use of BMSC-derived tissues in treating cartilage defect repair, and these obstacles need to be resolved definitively prior to clinical application [7, 36]. The broad efforts to develop new technologies and clinical trials to repair cartilage using cell-based products are reviewed here [49–51].

## Conclusion

In summary, cell-mediated cartilage repair strategies in the leading large animal cartilage repair model, the sheep, remain in their infancy. Identifying optimal oBMSC culture conditions and understanding the limitations of oBMSC will likely be critical to advancing the field of cartilage defect repair. Our data demonstrate that *(1)* there is limited cross-reactivity between common hBMSC antibody panels and oBMSC, but that CD44, CD146, and CD45 are useful markers; *(2)* IBMX is toxic during oBMSC adipogenic culture, but IBMX is not required and adipogenic induction can be salvaged by excluding this molecule from the medium; *(3)* oBMSC chondrogenesis can be enhanced by using micro-pellet cultures and reduced oxygen atmospheres in vitro; *(4)* micro-pellets formed from oBMSC appear morphologically similar to micro-pellets formed from oACh, but have upregulated hypertrophic gene expression in vitro; and *(5)* oBMSC micro-pellets produce inferior cartilage repair tissue in vivo, compared to oACh micro-pellets.

## Supplementary information

**Additional file 1: Supplementary Figure 1.** Flow cytometry analysis of cell surface markers for oBMSC 1.

**Additional file 2: Supplementary Figure 2.** Flow cytometry analysis of cell surface markers for oBMSC 2.

**Additional file 3: Supplementary Figure 3.** Flow cytometry analysis of cell surface markers for oBMSC 3.

**Additional file 4: Supplementary Figure 4.** Control flow cytometry analysis of cell surface markers for fresh sheep mononuclear cells (MNC) isolated from sheep bone marrow aspirate.

**Additional file 5: Supplementary Figure 5.** Control flow cytometry analysis of cell surface markers on expanded hBMSC.

**Additional file 6: Supplementary Figure 6.** Microscopic images over a 14-day culture period of two additional oBMSC donors cultured as *macro*-pellets in deep-well plates (A and C), and cultured as *micro*-pellets in the *Microwell-mesh* platform (B and D). Scale bar = 1 mm.

**Additional file 7: Supplementary Figure 7.** Type II collagen staining in A) *macro*-pellet and B) *micro*-pellet sections. Scale bar = 400 μm.

**Additional file 8: Supplementary Figure 8.** Type I collagen staining in A) *macro*-pellet and B) *micro*-pellet sections. Scale bar = 400 μm.

**Additional file 9: Supplementary Figure 9.** Type X collagen staining in A) *macro*-pellet and B) *micro*-pellet sections. Scale bar = 400 μm.

**Additional file 10: Supplementary Figure 10.** Scaffold-only controls for A) oACh sheep pilot, and B) oBMSC sheep pilot. The middle and right images are enlarged from the boxes indicated in the images on the left. The * points to non-degraded scaffold, and the triangle points to a blood vessel. Tissue was fibrous with negligible repair.

**Additional file 11: Supplementary Figure 11.** Photos of joints 8 weeks after surgery. Asterisk indicates scaffold-only controls. Arrow points to the best repair observed with oACh *micro*-pellets.

## Data Availability

All data is available through the senior author.

## Abbreviations

ACAN: Aggrecan
ANOVA: Analysis of variance
Anti-Anti: Antibiotic-Antimycotic
BMP2: Bone Morphogenetic Protein 2
BMSC: Bone marrow stromal cells
BSA: Bovine serum albumin
COL10A1: Collagen Type X Alpha 1 Chain
COL1A1: Collagen Type I Alpha 1 Chain
COL2A1: Collagen Type II Alpha 1 Chain
Dex: Dexamethasone
DMSO: Dimethyl sulfoxide
DNA: Deoxyribonucleic acid
dsDNA: Double stranded deoxyribonucleic acid
ECM: Extracellular matrix
EDTA: Ethylenediaminetetraacetic acid
GAG: Glycosaminoglycan
GAPDH: Glyceraldehyde 3-phosphate dehydrogenase
H&E: Haematoxylin and eosin
hBMSC: Human bone marrow stromal cells
HG-DMEM: High glucose Dulbecco’s modified Eagle’s medium
IBMX: 3-Isobutyl-1-methylxanthine
LG-DMEM: Low glucose Dulbecco’s modified Eagle’s medium
MNC: Mononuclear cells
oACH: Ovine articular chondrocytes
oBMSC: Ovine bone marrow stromal cells
PBS: Phosphate-buffered saline
PDMS: Polydimethylsiloxane
PenStrep: Penicillin/streptomycin
PFA: Paraformaldehyde
qPCR: Quantitative polymerase chain reaction
QUT: Queensland University of Technology
RNase: Ribonuclease
RUNX2: RUNX Family Transcription Factor 2
SOX9: SRY-Box Transcription Factor 9
WNT11: Wnt family member 11

## References

[1] Negoro T, Takagaki Y, Okura H, Matsuyama A (2018). Trends in clinical trials for articular cartilage repair by cell therapy. NPJ Regen Med.

[2] Serafini M, Sacchetti B, Pievani A, Redaelli D, Remoli C, Biondi A, Riminucci M, Bianco P (2014). Establishment of bone marrow and hematopoietic niches in vivo by reversion of chondrocyte differentiation of human bone marrow stromal cells. Stem Cell Res.

[3] Futrega K, Palmer JS, Kinney M, Lott WB, Ungrin MD, Zandstra PW, Doran MR (2015). The microwell-mesh: a novel device and protocol for the high throughput manufacturing of cartilage microtissues. Biomaterials.

[4] Cook JL, Hung CT, Kuroki K, Stoker AM, Cook CR, Pfeiffer FM, Sherman SL, Stannard JP (2014). Animal models of cartilage repair. Bone Joint Res.

[5] Moran CJ, Ramesh A, Brama PA, O'Byrne JM, O'Brien FJ, Levingstone TJ (2016). The benefits and limitations of animal models for translational research in cartilage repair. J Exp Orthop.

[6] Music E, Futrega K, Doran MR (2018). Sheep as a model for evaluating mesenchymal stem/stromal cell (MSC)-based chondral defect repair. Osteoarthr Cartil.

[7] Futrega K, Robey PG, Klein TJ, Crawford RW, Doran MR. Micro-pellet culture reveals that bone marrow mesenchymal stromal cell (BMSC) chondrogenic induction is triggered by a single day of TGF-beta1 exposure. bioRxiv. 2019. 10.1101/853556.

[8] Markway BD, Tan GK, Brooke G, Hudson JE, Cooper-White JJ, Doran MR (2010). Enhanced chondrogenic differentiation of human bone marrow-derived mesenchymal stem cells in low oxygen environment micropellet cultures. Cell Transplant.

[9] Johnstone B, Hering TM, Caplan AI, Goldberg VM, Yoo JU (1998). In vitro chondrogenesis of bone marrow-derived mesenchymal progenitor cells. Exp Cell Res.

[10] Babur BK, Futrega K, Lott WB, Klein TJ, Cooper-White J, Doran MR (2015). High-throughput bone and cartilage micropellet manufacture, followed by assembly of micropellets into biphasic osteochondral tissue. Cell Tissue Res.

[11] Murphy CL, Thoms BL, Vaghjiani RJ, Lafont JE (2009). Hypoxia. HIF-mediated articular chondrocyte function: prospects for cartilage repair. Arthritis Res Ther.

[12] Thoms BL, Dudek KA, Lafont JE, Murphy CL (2013). Hypoxia promotes the production and inhibits the destruction of human articular cartilage. Arthritis Rheum.

[13] Robins JC, Akeno N, Mukherjee A, Dalal RR, Aronow BJ, Koopman P, Clemens TL (2005). Hypoxia induces chondrocyte-specific gene expression in mesenchymal cells in association with transcriptional activation of Sox9. Bone.

[14] Berner A, Henkel J, Woodruff MA, Saifzadeh S, Kirby G, Zaiss S, Gohlke J, Reichert JC, Nerlich M, Schuetz MA, Hutmacher DW (2017). Scaffold-cell bone engineering in a validated preclinical animal model: precursors vs differentiated cell source. J Tissue Eng Regen Med.

[15] Ungrin MD, Joshi C, Nica A, Bauwens C, Zandstra PW (2008). Reproducible, ultra high-throughput formation of multicellular organization from single cell suspension-derived human embryonic stem cell aggregates. PLoS One.

[16] Chambers KF, Mosaad EM, Russell PJ, Clements JA, Doran MR (2014). 3D cultures of prostate cancer cells cultured in a novel high-throughput culture platform are more resistant to chemotherapeutics compared to cells cultured in monolayer. PLoS One.

[17] Bianco P, Robey PG, Saggio I, Riminucci M (2010). “Mesenchymal” stem cells in human bone marrow (skeletal stem cells): a critical discussion of their nature, identity, and significance in incurable skeletal disease. Hum Gene Ther.

[18] Deschaseaux F, Gindraux F, Saadi R, Obert L, Chalmers D, Herve P (2003). Direct selection of human bone marrow mesenchymal stem cells using an anti-CD49a antibody reveals their CD45med,low phenotype. Br J Haematol.

[19] Siddappa R, Licht R, van Blitterswijk C, de Boer J (2007). Donor variation and loss of multipotency during in vitro expansion of human mesenchymal stem cells for bone tissue engineering. J Orthop Res.

[20] Dominici M, Le Blanc K, Mueller I, Slaper-Cortenbach I, Marini F, Krause D, Deans R, Keating A, Prockop D, Horwitz E (2006). Minimal criteria for defining multipotent mesenchymal stromal cells. The International Society for Cellular Therapy position statement. Cytotherapy.

[21] Sorrentino A, Ferracin M, Castelli G, Biffoni M, Tomaselli G, Baiocchi M, Fatica A, Negrini M, Peschle C, Valtieri M (2008). Isolation and characterization of CD146+ multipotent mesenchymal stromal cells. Exp Hematol.

[22] Sacchetti B, Funari A, Michienzi S, Di Cesare S, Piersanti S, Saggio I, Tagliafico E, Ferrari S, Robey PG, Riminucci M, Bianco P (2007). Self-renewing osteoprogenitors in bone marrow sinusoids can organize a hematopoietic microenvironment. Cell.

[23] Shi S, Gronthos S (2003). Perivascular niche of postnatal mesenchymal stem cells in human bone marrow and dental pulp. J Bone Miner Res.

[24] Gronthos S, Simmons PJ (1995). The growth factor requirements of STRO-1-positive human bone marrow stromal precursors under serum-deprived conditions in vitro. Blood.

[25] Petters O, Schmidt C, Thuemmler C, Peinemann F, Zscharnack M, Somerson JS, Schulz RM (2018). Point-of-care treatment of focal cartilage defects with selected chondrogenic mesenchymal stromal cells - an in vitro proof-of-concept study. J Tissue Eng Regen Med.

[26] Barilani M, Banfi F, Sironi S, Ragni E, Guillaumin S, Polveraccio F, Rosso L, Moro M, Astori G, Pozzobon M, Lazzari L (2018). Low-affinity nerve growth factor receptor (CD271) heterogeneous expression in adult and fetal mesenchymal stromal cells. Sci Rep.

[27] Sanjurjo-Rodriguez C, Castro-Vinuelas R, Hermida-Gomez T, Fernandez-Vazquez T, Fuentes-Boquete IM, de Toro-Santos FJ, Diaz-Prado SM, Blanco-Garcia FJ (2017). Ovine mesenchymal stromal cells: morphologic, phenotypic and functional characterization for osteochondral tissue engineering. PLoS One.

[28] McCarty RC, Gronthos S, Zannettino AC, Foster BK, Xian CJ (2009). Characterisation and developmental potential of ovine bone marrow derived mesenchymal stem cells. J Cell Physiol.

[29] Essayan DM (2001). Cyclic nucleotide phosphodiesterases. J Allergy Clin Immunol.

[30] Scott MA, Nguyen VT, Levi B, James AW (2011). Current methods of adipogenic differentiation of mesenchymal stem cells. Stem Cells Dev.

[31] Rentsch C, Hess R, Rentsch B, Hofmann A, Manthey S, Scharnweber D, Biewener A, Zwipp H (2010). Ovine bone marrow mesenchymal stem cells: isolation and characterization of the cells and their osteogenic differentiation potential on embroidered and surface-modified polycaprolactone-co-lactide scaffolds. In Vitro Cell Dev Biol Anim.

[32] Sales VL, Mettler BA, Lopez-Ilasaca M, Johnson JA, Mayer JE (2007). Endothelial progenitor and mesenchymal stem cell-derived cells persist in tissue-engineered patch in vivo: application of green and red fluorescent protein-expressing retroviral vector. Tissue Eng.

[33] Krinner A, Zscharnack M, Bader A, Drasdo D, Galle J (2009). Impact of oxygen environment on mesenchymal stem cell expansion and chondrogenic differentiation. Cell Prolif.

[34] Zscharnack M, Poesel C, Galle J, Bader A (2009). Low oxygen expansion improves subsequent chondrogenesis of ovine bone-marrow-derived mesenchymal stem cells in collagen type I hydrogel. Cells Tissues Organs.

[35] Armoiry X, Cummins E, Connock M, Metcalfe A, Royle P, Johnston R, Rodrigues J, Waugh N, Mistry H (2019). Autologous chondrocyte implantation with chondrosphere for treating articular cartilage defects in the knee: an evidence review group perspective of a NICE single technology appraisal. Pharmacoeconomics.

[36] Somoza RA, Welter JF, Correa D, Caplan AI (2014). Chondrogenic differentiation of mesenchymal stem cells: challenges and unfulfilled expectations. Tissue Eng Part B Rev.

[37] Robey PG, Kuznetsov SA, Riminucci M, Bianco P (2014). Bone marrow stromal cell assays: in vitro and in vivo. Methods Mol Biol.

[38] Edwards PK, Ackland T, Ebert JR (2014). Clinical rehabilitation guidelines for matrix-induced autologous chondrocyte implantation on the tibiofemoral joint. J Orthop Sports Phys Ther.

[39] Gupta PK, Das AK, Chullikana A, Majumdar AS. Mesenchymal stem cells for cartilage repair in osteoarthritis. Stem Cell Res Ther. 2012;3(4):25. 10.1186/scrt116. 10.1186/scrt116 PMC3580463 22776206

[40] Bianco P, Robey PG, Simmons PJ (2008). Mesenchymal stem cells: revisiting history, concepts, and assays. Cell Stem Cell.

[41] McCarty RC, Xian CJ, Gronthos S, Zannettino AC, Foster BK (2010). Application of autologous bone marrow derived mesenchymal stem cells to an ovine model of growth plate cartilage injury. Open Orthop J.

[42] Jones CW, Willers C, Keogh A, Smolinski D, Fick D, Yates PJ, Kirk TB, Zheng MH (2008). Matrix-induced autologous chondrocyte implantation in sheep: objective assessments including confocal arthroscopy. J Orthop Res.

[43] Bozkurt M, Aşık MD, Gürsoy S, Türk M, Karahan S, Gümüşkaya B, Akkaya M, Şimşek ME, Cay N, Doğan M. Autologous stem cell-derived chondrocyte implantation with bio-targeted microspheres for the treatment of osteochondral defects. Orthop Surg Res. 2019;14:394. 10.1186/s13018-019-1434-0. 10.1186/s13018-019-1434-0 PMC6883666 31779662

[44] Dorotka R, Bindreiter U, Macfelda K, Windberger U, Nehrer S (2005). Marrow stimulation and chondrocyte transplantation using a collagen matrix for cartilage repair. Osteoarthr Cartil.

[45] Kaback LA, Soung do Y, Naik A, Smith N, Schwarz EM, O'Keefe RJ, Drissi H (2008). Osterix/Sp7 regulates mesenchymal stem cell mediated endochondral ossification. J Cell Physiol.

[46] Rashid H, Ma C, Chen H, Wang H, Hassan MQ, Sinha K, de Crombrugghe B, Javed A (2014). Sp7 and Runx2 molecular complex synergistically regulate expression of target genes. Connect Tissue Res.

[47] Occhetta P, Pigeot S, Rasponi M, Dasen B, Mehrkens A, Ullrich T, Kramer I, Guth-Gundel S, Barbero A, Martin I (2018). Developmentally inspired programming of adult human mesenchymal stromal cells toward stable chondrogenesis. Proc Natl Acad Sci U S A.

[48] Diederichs S, Tonnier V, Marz M, Dreher SI, Geisbusch A, Richter W (2019). Regulation of WNT5A and WNT11 during MSC in vitro chondrogenesis: WNT inhibition lowers BMP and hedgehog activity, and reduces hypertrophy. Cell Mol Life Sci.

[49] Arshi A, Petrigliano FA, Williams RJ, Jones KJ (2020). Stem cell treatment for knee articular cartilage defects and osteoarthritis. Curr Rev Musculoskelet Med.

[50] Lam ATL, Reuveny S, Oh SK-W. Human mesenchymal stem cell therapy for cartilage repair: review on isolation, expansion, and constructs. Stem Cell Res. 2020;44:101738. 10.1016/j.scr.2020.101738. 10.1016/j.scr.2020.101738 32109723

[51] Eder C, Schmidt-Bleek K, Geissler S, Sass FA, Maleitzke T, Pumberger M, Perka C, Duda GN, Winkler T (2020). Mesenchymal stromal cell and bone marrow concentrate therapies for musculoskeletal indications: a concise review of current literature. Mol Biol Rep.

