# Supplementary figures and images for: Characterisation of ovine bone marrow-derived stromal cells (oBMSC) and evaluation of chondrogenically induced micro-pellets for cartilage tissue repair in vivo
Source: Stem Cell Res Ther. 2021 Jan 7;12:26. doi: 10.1186/s13287-020-02045-3 (PMC7791713; doi:10.1186/s13287-020-02045-3)

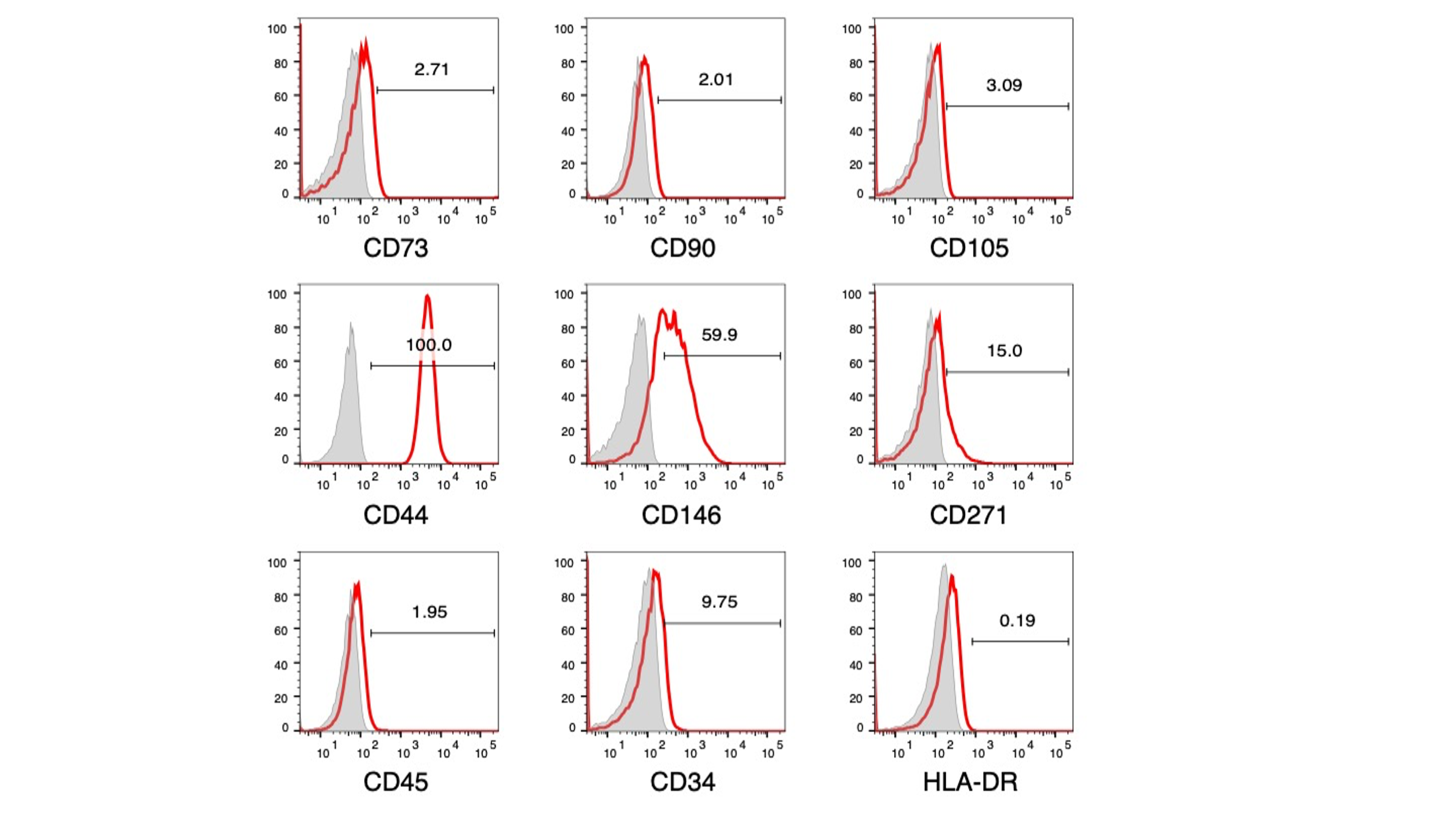

Supplement: Supplementary file 1 — Additional file 1: Supplementary Figure 1. Flow cytometry analysis of cell surface markers for oBMSC 1. [file 13287_2020_2045_MOESM1_ESM.tiff]

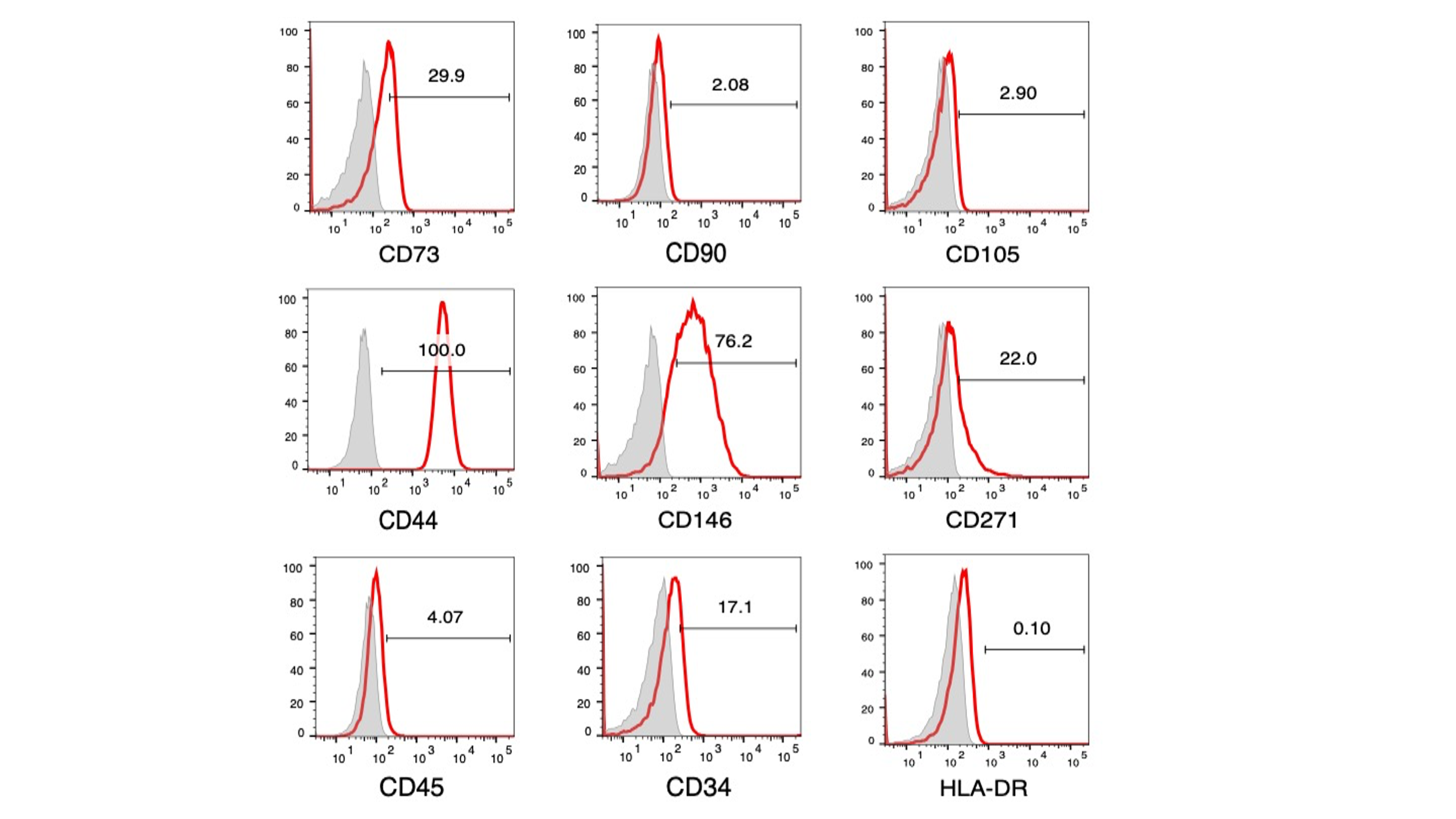

Supplement: Supplementary file 2 — Additional file 2: Supplementary Figure 2. Flow cytometry analysis of cell surface markers for oBMSC 2. [file 13287_2020_2045_MOESM2_ESM.tiff]

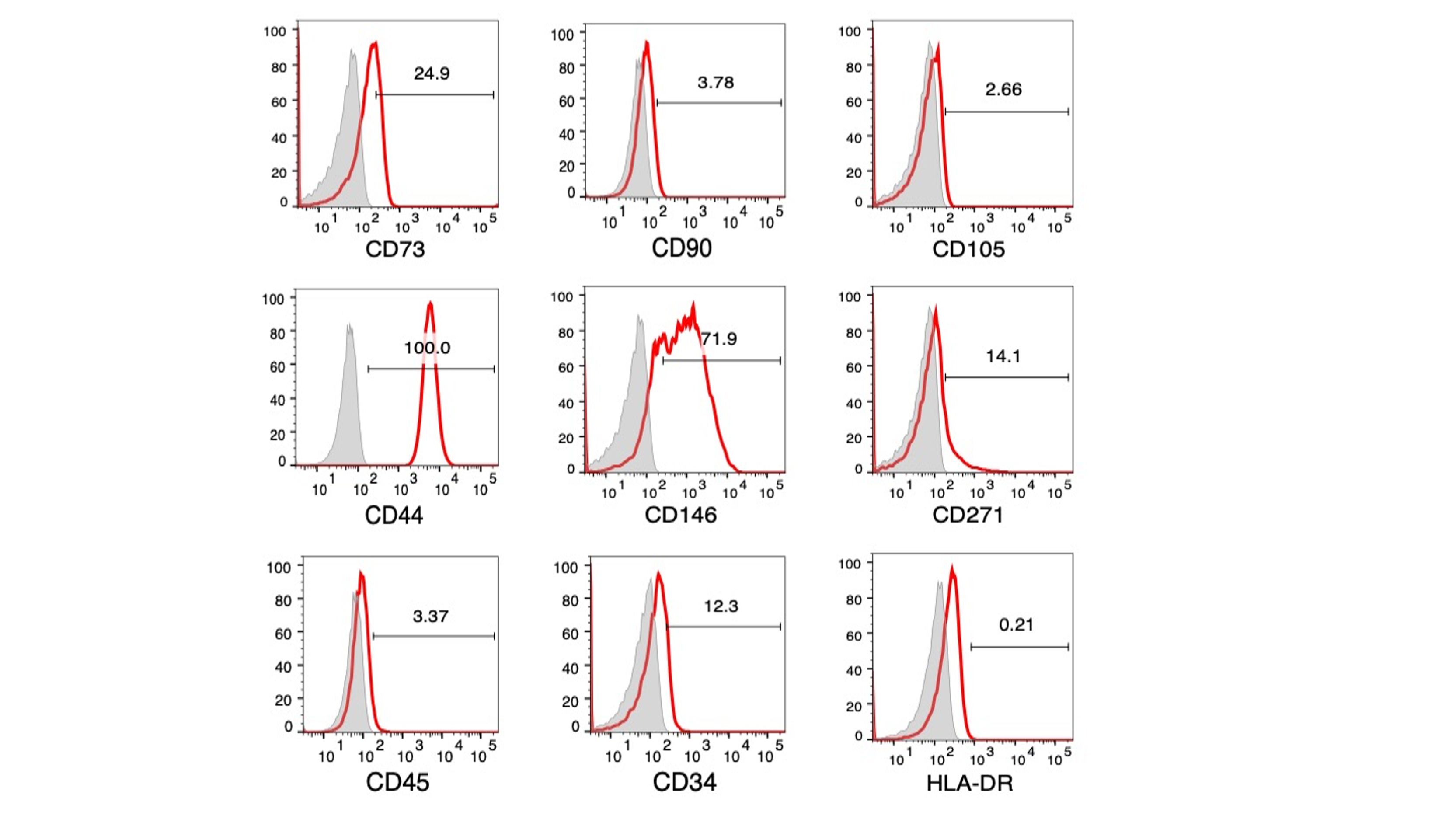

Supplement: Supplementary file 3 — Additional file 3: Supplementary Figure 3. Flow cytometry analysis of cell surface markers for oBMSC 3. [file 13287_2020_2045_MOESM3_ESM.tiff]

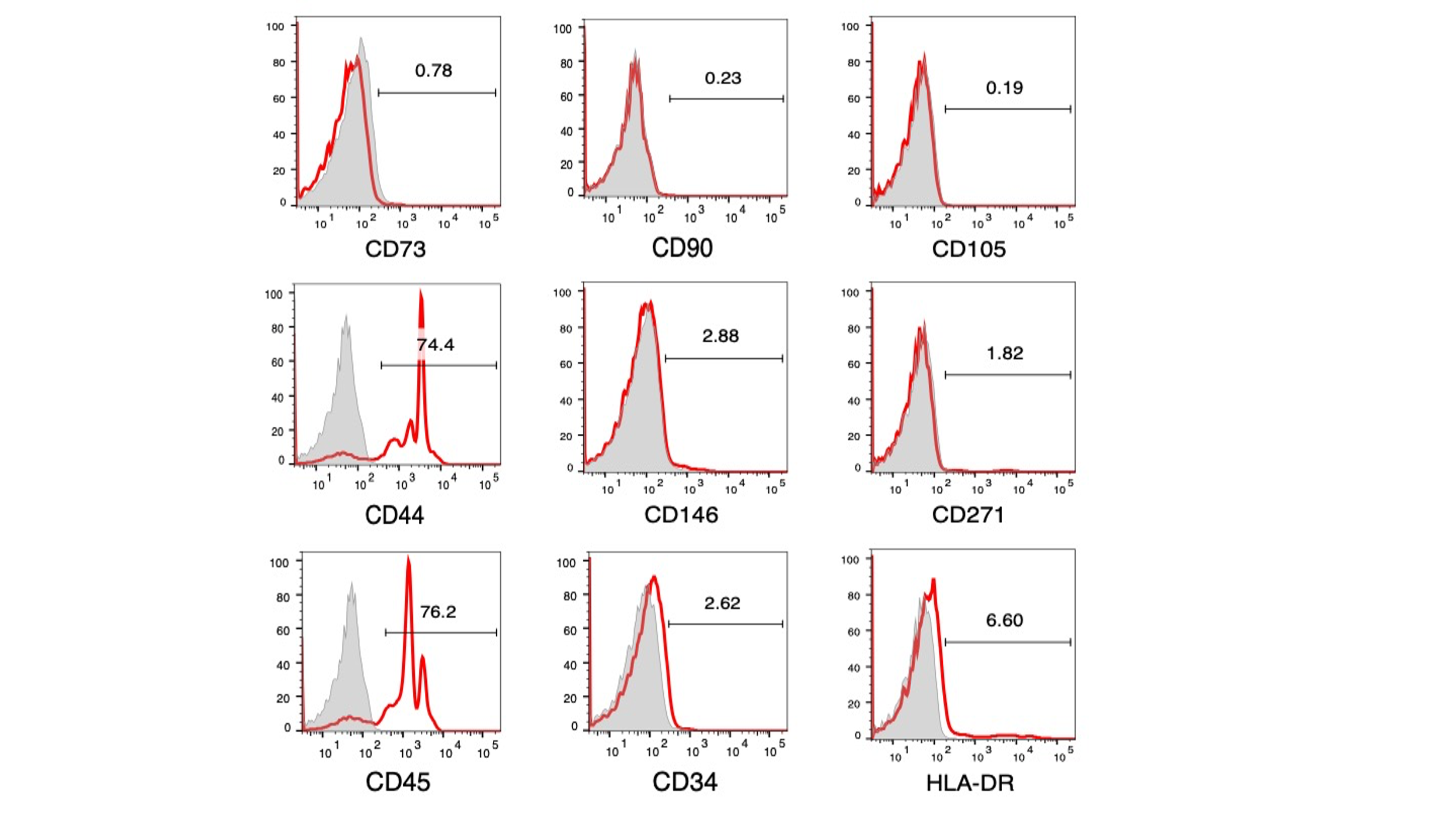

Supplement: Supplementary file 4 — Additional file 4: Supplementary Figure 4. Control flow cytometry analysis of cell surface markers for fresh sheep mononuclear cells (MNC) isolated from sheep bone marrow aspirate. [file 13287_2020_2045_MOESM4_ESM.tiff]

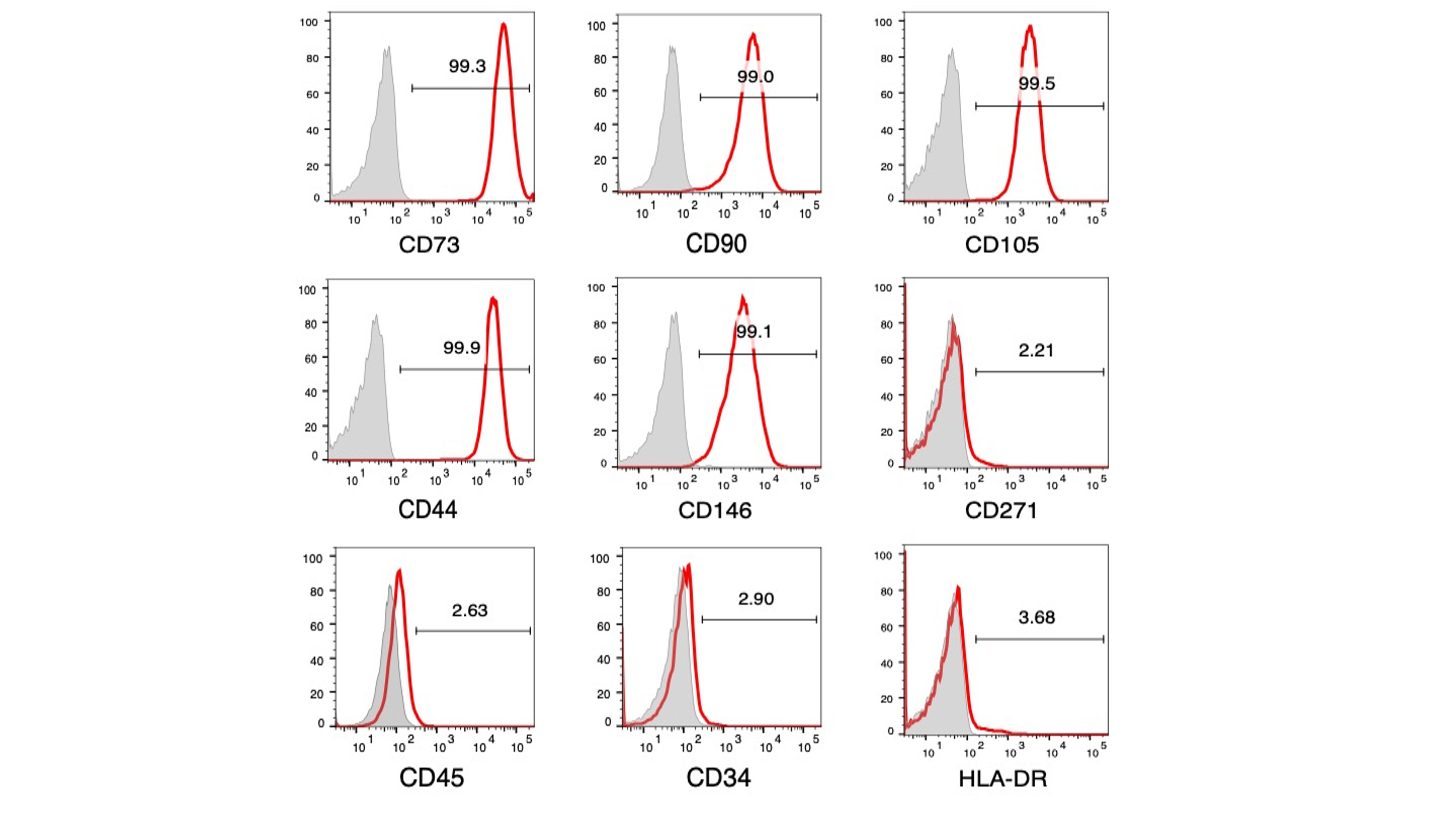

Supplement: Supplementary file 5 — Additional file 5: Supplementary Figure 5. Control flow cytometry analysis of cell surface markers on expanded hBMSC. [file 13287_2020_2045_MOESM5_ESM.tiff]

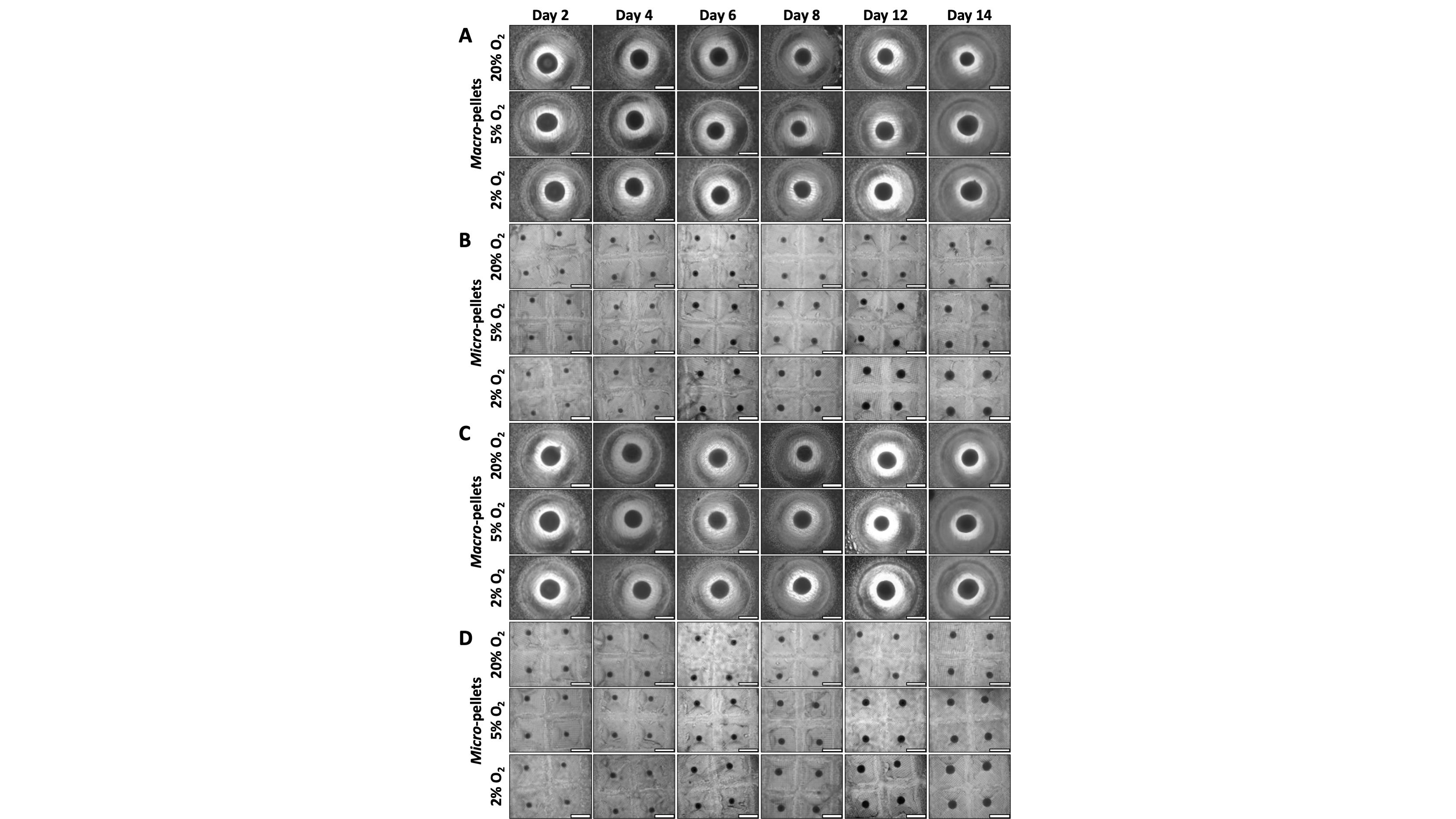

Supplement: Supplementary file 6 — Additional file 6: Supplementary Figure 6. Microscopic images over a 14-day culture period of two additional oBMSC donors cultured as macro-pellets in deep-well plates (A and C), and cultured as micro-pellets in the Microwell-mesh platform (B and D). Scale bar = 1 mm. [file 13287_2020_2045_MOESM6_ESM.tiff]

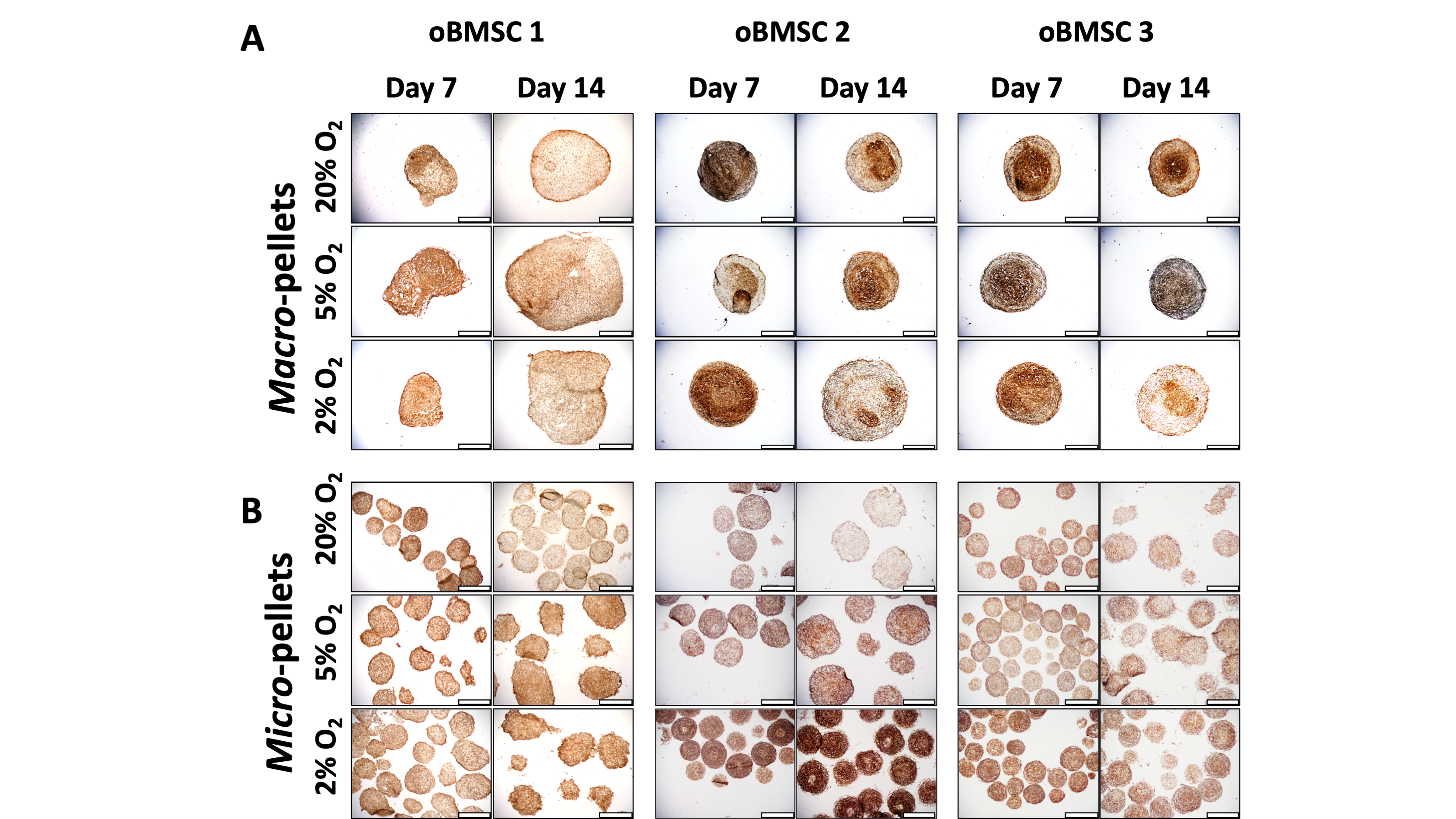

Supplement: Supplementary file 7 — Additional file 7: Supplementary Figure 7. Type II collagen staining in A) macro-pellet and B) micro-pellet sections. Scale bar = 400 μm. [file 13287_2020_2045_MOESM7_ESM.tiff]

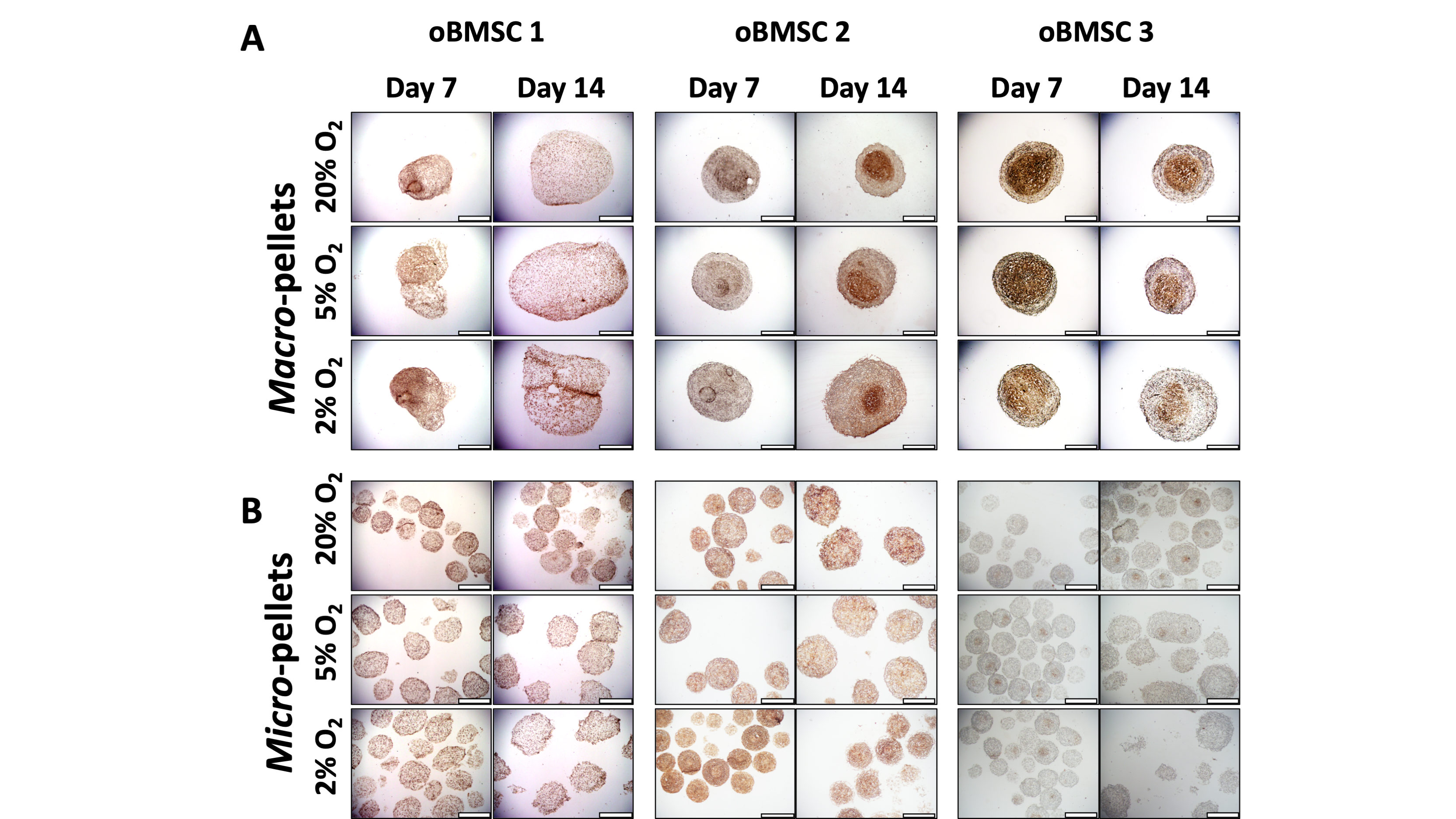

Supplement: Supplementary file 8 — Additional file 8: Supplementary Figure 8. Type I collagen staining in A) macro-pellet and B) micro-pellet sections. Scale bar = 400 μm. [file 13287_2020_2045_MOESM8_ESM.tiff]

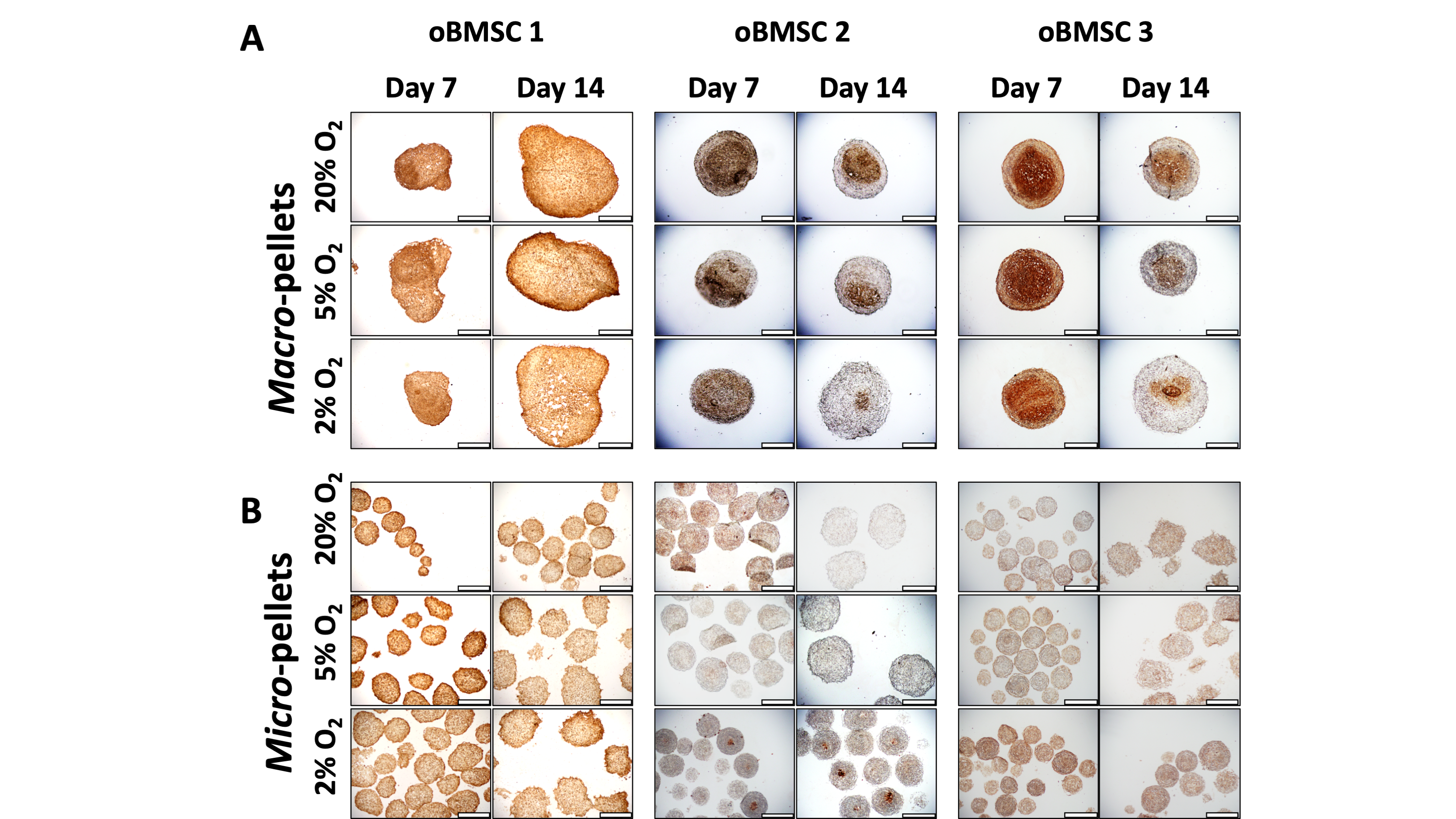

Supplement: Supplementary file 9 — Additional file 9: Supplementary Figure 9. Type X collagen staining in A) macro-pellet and B) micro-pellet sections. Scale bar = 400 μm. [file 13287_2020_2045_MOESM9_ESM.tiff]

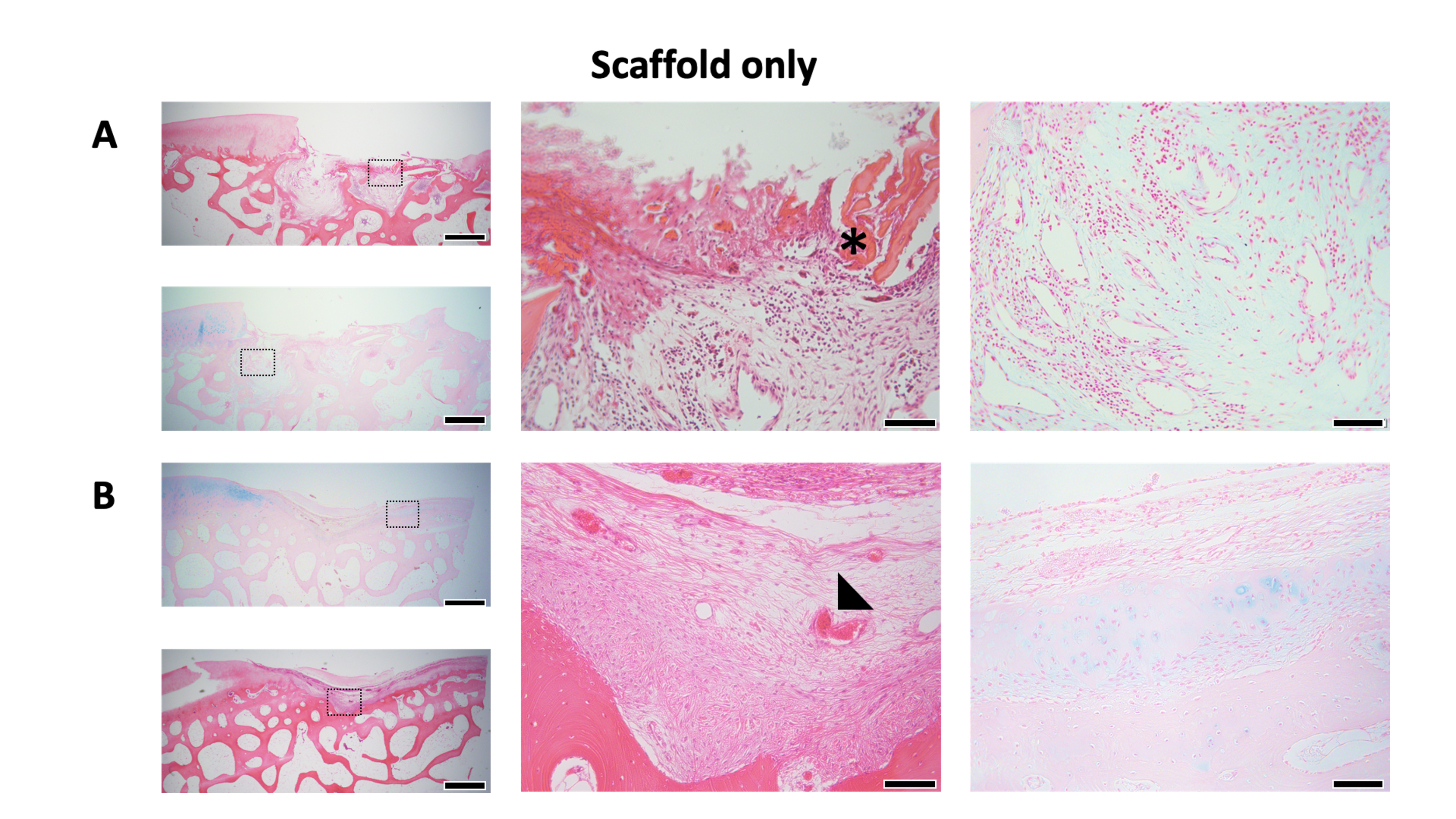

Supplement: Supplementary file 10 — Additional file 10: Supplementary Figure 10. Scaffold-only controls for A) oACh sheep pilot, and B) oBMSC sheep pilot. The middle and right images are enlarged from the boxes indicated in the images on the left. The * points to non-degraded scaffold, and the triangle points to a blood vessel. Tissue was fibrous with negligible repair. [file 13287_2020_2045_MOESM10_ESM.tiff]

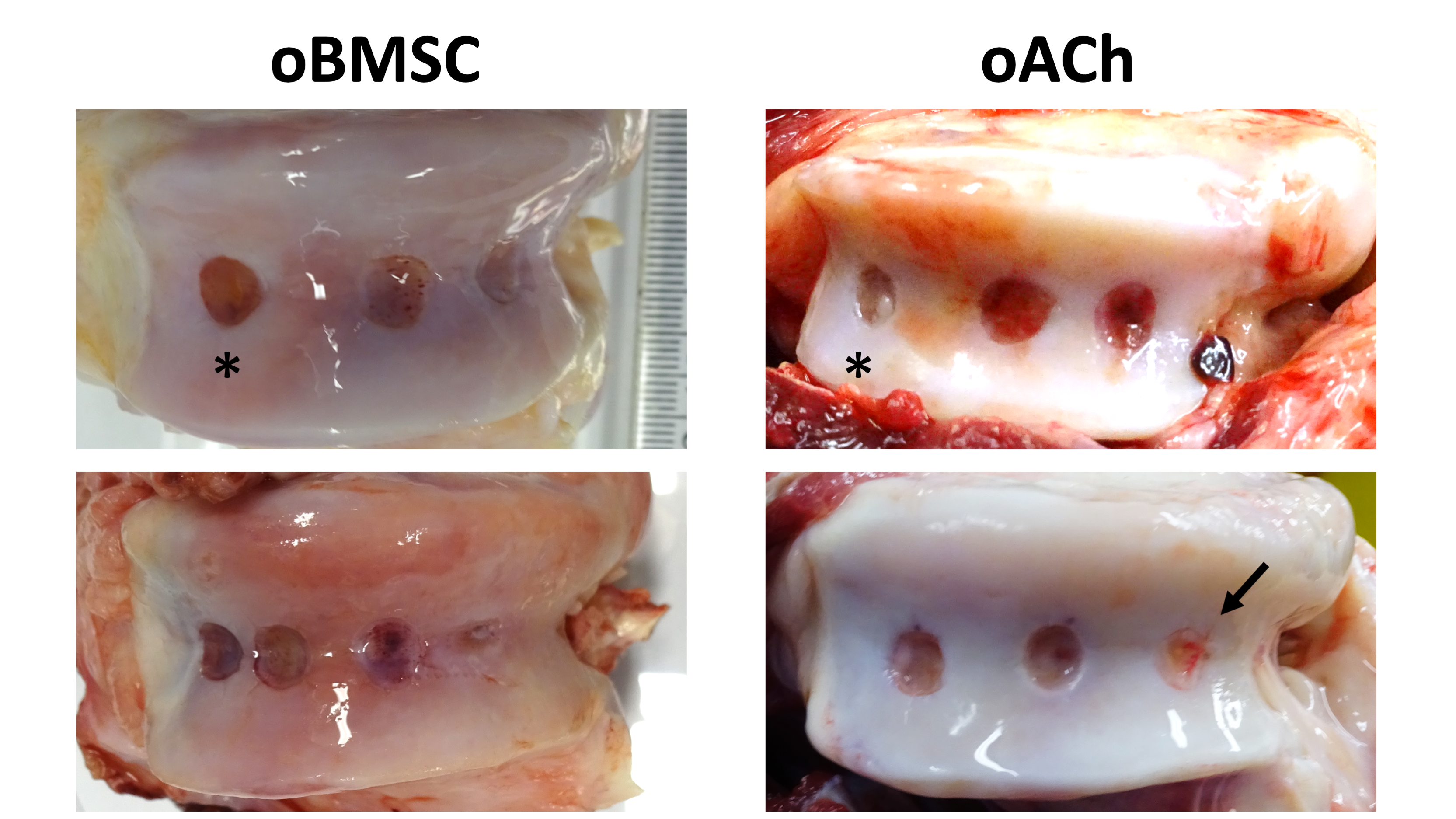

Supplement: Supplementary file 11 — Additional file 11: Supplementary Figure 11. Photos of joints 8 weeks after surgery. Asterisk indicates scaffold-only controls. Arrow points to the best repair observed with oACh micro-pellets. [file 13287_2020_2045_MOESM11_ESM.tiff]
